# The Belt and Road Initiative’s impact on tourism and heritage along the Silk Roads: A systematic literature review and future research agenda

**DOI:** 10.1371/journal.pone.0306298

**Published:** 2024-07-18

**Authors:** Bashar Dayoub, Peifeng Yang, Sarah Omran, Qiuyi Zhang, Xu Chen, Akram Ahmed Noman Alabsi, Alaa Dayoub

**Affiliations:** 1 College of Architecture and Urban Planning, Fujian University of Technology, Fuzhou, China; 2 Department of International Trade, China Financial Engineering Company Limited, Hong Kong, China; University of Kragujevac: Univerzitet u Kragujevcu, SERBIA

## Abstract

This is a systematic review of the literature on the Belt and Road Initiative (BRI) and its impact on tourism and heritage in participating countries along the Silk Roads. China launched the BRI in 2013 with the aim of promoting global trade and stimulating economic growth through the development of infrastructure and cultural cooperation. This review examines studies for the period from 2013 to 2023, focusing on key themes such as tourist flows, destination development, urban renewal, heritage preservation, and cultural route revival. The systematic review follows the Preferred Reporting Items for Systematic Reviews and Meta-Analyses (PRISMA) guidelines, incorporating 56 relevant documents that cover both tourism and heritage domains. The findings highlight substantial potential for the development of new tourism products and destinations, improved urban renewal, and the preservation of cultural heritage, provided that integrated policies, public-private collaboration, and equitable community participation frameworks are implemented with attention to ecological limits. However, the review also identifies significant challenges, including financial imbalances, uneven access to benefits, social disruption, cultural commodification, and environmental degradation. Addressing these issues requires careful, context-specific planning. The study concludes with a proposal for a future research agenda that includes exploring underrepresented regions, developing sustainable tourism models, and fostering interdisciplinary research to ensure a balanced approach to economic development and heritage preservation. This review’s findings provide valuable insight for policymakers, tourism officials, and cultural heritage managers, guiding the development of policies that balance economic growth with the preservation of cultural and natural heritage sites. This research contributes to the academic discourse by elucidating the complex interplay between the BRI and the Silk Roads’ tourism and heritage, offering a pathway for sustainable and inclusive growth.

## 1 Introduction

The Belt and Road Initiative (BRI) launched by China in 2013 aims to enhance global trade and stimulate economic growth across Asia and beyond by developing trade routes reminiscent of the ancient Silk Road. This initiative has significant implications for infrastructure development, tourism, and local communities along the Silk Roads. The BRI includes the Silk Road Economic Belt and the 21st Century Maritime Silk Road, focusing on infrastructure investment, financial integration, trade facilitation, policy coordination, and cultural cooperation among its participating countries [1–4]. Economically, the BRI fosters connectivity and trade through significant infrastructure projects such as railways, ports, and highways. The aim of these projects is to stimulate economic growth and development in participating countries. For instance, the China-Pakistan Economic Corridor (CPEC) includes extensive infrastructure investments to boost trade and tourism [5]. This initiative has received significant attention from the international academic community, with a multitude of studies examining it from various angles, and exploring its array of impacts [6–9].

The Silk Road, an ancient network of land and sea routes connecting the East and the West, has played a crucial role in human history, facilitating the exchange of goods, ideas, and cultural values [10–14]. German geographer Ferdinand von Richthofen coined the term "Silk Road" in 1877 to recognize the historical importance of this network and its associated settlements [15]. Besides serving as a trade route, the Silk Road facilitated cultural exchange, enabling the spread of literature, knowledge, art, beliefs, and religion among disparate groups of people [16–18].

One aim of the BRI is to boost tourism in countries along the Silk Road and attract tourists from these countries to new destinations [19–23]. However, a balance must be struck between economic development, heritage preservation, and sustainable practices [24–27]. International organizations such as UNESCO, UNWTO, and the EU have supported various Silk Road projects, highlighting the Silk Road’s tourism potential and cultural significance [28, 29].

The BRI’s impact on tourism development and the quality of life for residents is notable, as new infrastructure opportunities foster an environment conducive to tourism, thereby improving residents’ quality of life [5]. Furthermore, the BRI acts as a catalyst for infrastructure development and generates substantial benefits for local communities, including employment opportunities and improved social services [30]. Additionally, the economic performance of some countries along the BRI during the COVID-19 pandemic demonstrates its resilience and importance in maintaining economic stability and fostering regional development [31].

The BRI’s routing is based on the Ancient Silk Road, and its economic focus has affected both tourism and cultural significance along the communities it connects [32, 33]. However, some observers believe that cultural considerations may have been overlooked in the early stages of developing the Silk Road Economic Belt [30]. In contrast, others believe cultural revival and heritage conservation will naturally arise from strengthening regional connections and economic progress [34]. Research on natural and cultural heritage conservation is ongoing, including efforts to preserve the Silk Road as a World Heritage Site and integrate it into tourism [35–37].

This study offers a significant contribution to the academic discourse by addressing the limited scholarly interest in the BRI’s impact on tourism and heritage along the historical Silk Roads. By employing a multifaceted analytical framework, we comprehensively examine the complex interplay between the BRI and the intricate tourism, history, culture, and economics of the Silk Roads. In the analysis, we evaluate the theoretical potential and practical obstacles associated with revitalizing this ancient corridor for contemporary tourism and heritage conservation efforts through meticulous case studies.

This study also outlines a comprehensive future research agenda, with emphasis on sustainable tourism practices, inclusive stakeholder engagement, economic viability, and heritage conservation. The methodological and analytical innovations embedded within the study’s framework pave the way for accurate understanding and equitable outcomes in tourism development.

By elucidating these complex interactions, this research also provides valuable visions for policymakers and researchers, and calls for continued scientific engagement, interdisciplinary cooperation, and international dialogue to ensure that the BRI promotes sustainable growth, cultural exchange, and heritage protection along this iconic global crossroads. Ultimately, our aim with this paper is to channel the ambitious Silk Road vision equitably towards empowering communities, stimulating connectivity, and upholding enduring values, as a path to modernization rooted in past legacies.

## 2 Overview of the study area

Most of the literature on the BRI discusses tourism issues specific to certain geographical areas of the Silk Road. Thus, reviewing the literature on the BRI and its impact on tourism and heritage along the Silk Road necessitates clarification of the Silk Road’s comprehensive geographical scope. This facilitates a connection between the context of the literature review and the regions and countries that will be discussed.

The BRI project includes the Maritime Silk Road (MSR), crossing the Indian Ocean, Red Sea, and Mediterranean Sea, as well as the Land Silk Road (LSR) crossing Central Asia, the Middle East, and Europe. As the cradles of ancient civilizations, Europe, Africa, and Asia, through which the LSR and MSR travel, are rich in cultural resources. Along the MSR and LSR are 776 heritage sites, including cultural, natural, and mixed heritage sites, representing 69.2% of total global heritage sites [38]. Due to its historical, cultural, and natural significance, this vast region is ideal for tourism research on the Silk Road [39–42].

The Ancient Silk Roads (see S1 Fig) spanned from China to Europe through Central and Western Asia [43, 44], connecting rich natural, cultural, and historical sites [45]. These sites, scattered throughout ancient trade routes, demonstrate the web of connections between the civilizations interacting along this corridor [46, 47]. The North Road, which runs through Samarkand and Ray on the Iranian plateau to the Mediterranean [48, 49], emerged as the principal artery of this complex network [25].

Besides the namesake Chinese silk textile exports to Roman markets hungry for luxuries, these premodern conduits facilitated regional travel and bartering of gems, spices, teas, medicine, textiles, metals, livestock, and even ideas and people [22, 50].

This cultural heritage shaped by centuries of pan-Eurasian exchange constitutes a powerful binding glue today that can help inspire cooperative regional ties and tourism to rediscover historical connections [51]. Several known Silk Road-themed circuits already attract visitors. Tourists traverse old caravan paths to explore the legacies of once-vibrant hubs like Xi’an, Samarkand, Kashgar, Persepolis, Istanbul, Mousel, Palmyra, Damascus, Tyre, Aleppo, and Antakya up close [52]. This interest looks likely to expand as more countries open up tourism infrastructure. However, limitations exist in fully optimizing the potential [22].

Potential and recognized world heritage sites and historic cities exemplify this ancient route’s shared history and cultural diversity [53, 54]. These sites include natural wonders, architectural marvels, and archaeological relics (see S2 Fig), providing unique tourism experiences, including adventure travel and cultural and natural heritage tourism [55]. As of this writing, regional and international efforts are being undertaken to protect the Silk Road’s architectural, cultural, and historical treasures [56–58]. The Silk Road’s regional history enhances its cultural exchanges by connecting more than 500 UNESCO World Heritage Sites across the Silk Roads [59].

The BRI/OBOR countries along the Ancient Silk Roads have also reported booming tourism revenues since the announcement of the BRI [60–63]. (S3 Fig) reveals the economic impact of tourism in the countries along the ancient Silk Roads by showing the tourism sector’s contribution to GDP [64]. The BRI prioritizes Silk Road tourism due to its historical significance and role in fostering interdependence and connecting tourism facilities, and thus, economic development [26].

## 3 Materials and methods

This study includes a systematic review of the literature on the Belt and Road Initiative (BRI) in tourism and heritage in BRI countries. The BRI countries were selected based on their geographical connectivity, cultural affinity with the Silk Road, and tourism potential. Numerous countries from across the globe are participating in the BRI. The Silk Routes are used as part of the research methodology because a dynamic of new tourism products and destinations is emerging from the revival of the Ancient Silk Road legacy.

**Fig 1:**
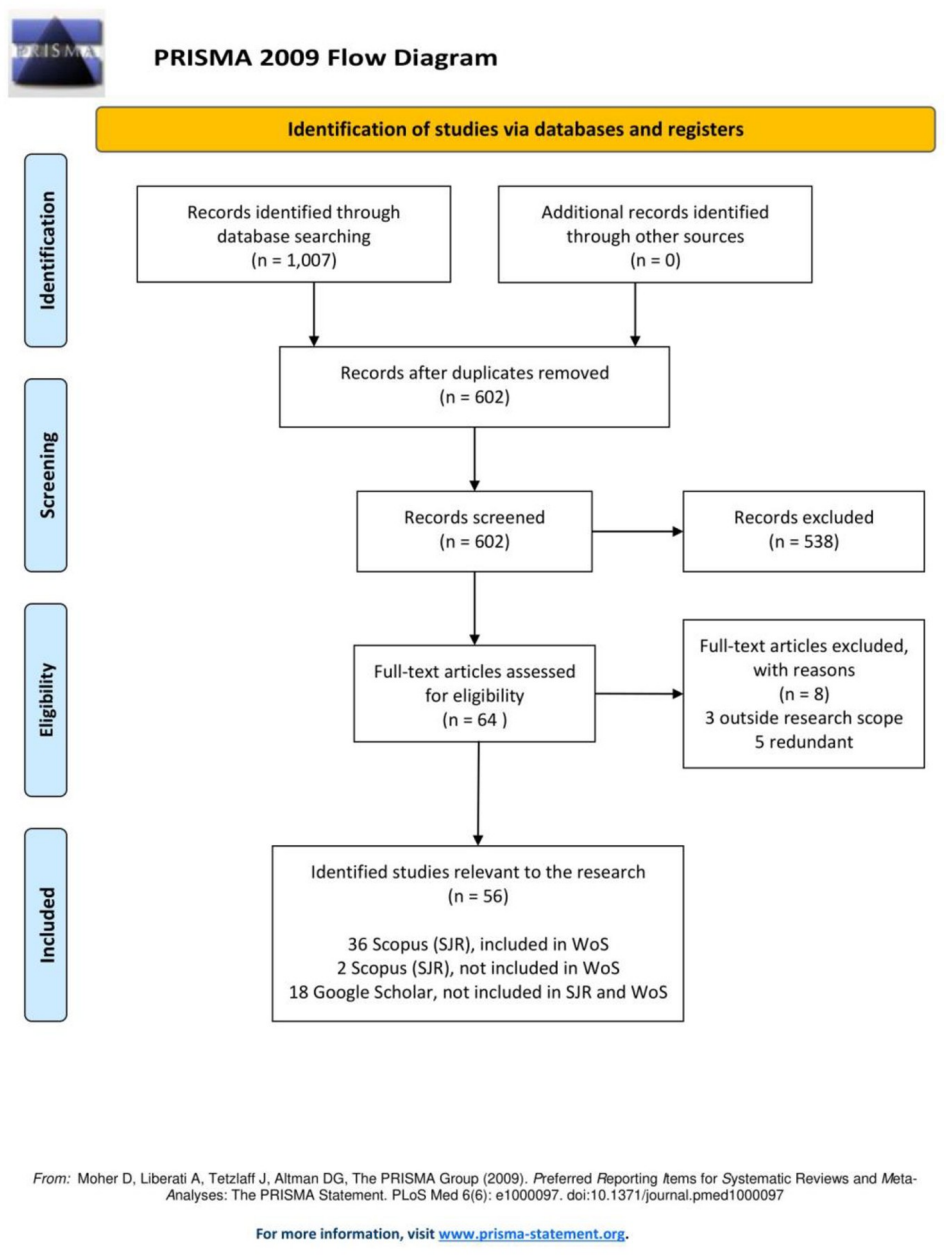
PRISMA 2009 flowchart of the literature review process. Source: edited by the authors.

The systematic review was conducted using the Preferred Reporting Items for Systematic Reviews and Meta-Analyses (PRISMA) guidelines (S1 Table). This review was not registered in Protocols.io or comparable registries. The study methodology adheres to PRISMA standards, as depicted in Fig 1. The aim of the review was to address the following:

*Core Research Question*

How does the Belt and Road Initiative (BRI) influence tourism and heritage along the Silk Roads?

*Research Objectives*

1. Comprehensive Survey of Existing BRI Research
  - Objective: To survey and review existing literature on the BRI’s impact on tourism and heritage
  - Scope: Identify relevant studies up to September 28, 2023.
  - Methodology: Systematic literature review using the PRISMA guidelines
2. Identification of Key Themes, Trajectories, and Gaps
  - Objective: Identify key themes, trajectories, and gaps in scholarly discourse on the BRI’s impact on tourism and heritage.
  - Scope: Categorize and analyze the reviewed literature to pinpoint common themes and research gaps.
  - Methodology: Narrative synthesis and bibliometric analysis using tools like Litmaps
3. Formulation of Recommendations for Future Research Directions
  - Objective: Formulate actionable recommendations for future research based on identified themes and gaps.
  - Scope: Propose new areas of investigation and methodologies to address the gaps identified.
  - Methodology: Expert consultation and synthesis of findings from the literature review

### 3.1 Eligibility criteria

Studies were included if they met the following criteria:

- Written in English
- Focused on the BRI and its impact on tourism and heritage along the Silk Roads
- Included peer-reviewed articles, reports from international organizations, and government documents

Studies were excluded if they were:

- Written in any language other than English
- Beyond the scope of tourism and heritage studies
- Irrelevant to heritage protection and tourism development
- Purely technical or methodological studies

### 3.2 Search strategy

We conducted a comprehensive literature search in Scopus, Web of Science, and Google Scholar for the period from 2013 to 2023. The search terms included: "Belt and Road Initiative," "BRI," "One Belt One Road," "OBOR," "B&R," "Silk Road," and "Silk Route." These terms were combined with keywords related to tourism and heritage (S2 and S3 Tables). The search was limited to articles, conference proceedings, and book chapters.

Acknowledging the dynamic nature of research in this domain, we conducted searches at multiple timepoints, culminating in the most recent search on September 28, 2023, to capture the latest scholarly contributions on the BRI’s impact on tourism and heritage.

### 3.3 Study selection

The study selection process involved three steps:

1. Duplicates were removed.
2. Titles and abstracts were screened independently by two reviewers.
3. Full texts of the remaining articles were assessed for eligibility.

Any disagreements were resolved through discussion or by involving a third author (P.Y.). The PRISMA flow diagram (Fig 1) illustrates the study selection process.

### 3.4 Data extraction

Two reviewers (B.D. and S.O.) extracted data independently and included information on study characteristics, BRI policies and projects, impact on tourism and heritage, and key findings. Any disagreements were resolved through discussion, or by involving a third author (P.Y.).

### 3.5 Quality assessment and risk of bias analysis

The quality of the included studies was assessed using the SCImago Journal Rank (SJR) and inclusion in the Scopus database (S2 Table), as well as their standing in Google Scholar (S3 Table). Most studies (68%) were published in journals indexed in both SJR and Scopus, while 32% were found in Google Scholar.

These studies were also assessed using the 2018 version of the Mixed Methods Appraisal Tool (MMAT) [65]. The MMAT evaluates the quality of empirical studies in systematic reviews, including qualitative, quantitative, and mixed-methods studies. Two authors (B.D. and S.O.) independently assessed the quality of each study, with any disagreements resolved through discussion, or the involvement of a third author (P.Y.). MMAT criteria were used to evaluate each study’s qualitative and quantitative components, and an overall quality score was assigned based on the number of criteria met. The quality assessment results for each study are presented in Table 1. The potential effect of study quality on the review results is discussed in the Discussion section, and sensitivity analyses were conducted to explore the results’ robustness to study quality.

**Table 1:** Quality assessment of included studies using the Mixed Methods Appraisal Tool (MMAT), version 2018. Source: edited by the authors.

Study	MMAT criteria met	MMAT Score (%)	Overall quality score	Risk of Bias	Notes
(Shymanskyi et al., 2022) [20]	4/5	80%	High	Low	Lack of clear information on the randomization process
(Deng and Hu, 2019) [26]	3/5	60%	Moderate	Moderate	Limited information on the sampling strategy and data analysis
(Li et al., 2021) [66]	5/5	100%	High	Low	Well-conducted study with clear reporting
(Kuchumov and Testina, 2020) [67]	3/5	60%	Moderate	Moderate	Insufficient details on the data collection process and potential confounders
(Raimkulov et al., 2021) [68]	4/5	80%	High	Low	Adequate reporting, but limited discussion of limitations
(Tikunov et al., 2017) [69]	2/5	40%	Low	High	Significant methodological issues and lack of transparency
(Juraturgunov et al., 2023) [70]	4/5	80%	High	Low	Well-conducted study, but small sample size
(Ali et al., 2017) [71]	3/5	60%	Moderate	Moderate	Limited information on the data analysis and potential bias
(Kučerová et al., 2020) [72]	4/5	80%	High	Low	Adequate reporting, but limited generalizability
(J Chen et al., 2021) [73]	5/5	100%	High	Low	Well-conducted study with clear reporting and robust methodology
(Idikut, 2020) [74]	3/5	60%	Moderate	Moderate	Limited information on the sampling strategy and potential confounders
(Y Chen et al., 2021) [45]	4/5	80%	High	Low	Adequate reporting, but limited discussion of limitations
(Chan et al., 2018) [16]	3/5	60%	Moderate	Moderate	Limited information on the data collection process and potential bias
(Daye et al., 2020) [75]	4/5	80%	High	Low	Well-conducted study, but small sample size
(Zhang et al., 2020) [76]	5/5	100%	100%	Low	Well-conducted study with clear reporting and robust methodology
(Mamirkulova et al., 2020) [5]	3/5	60%	Moderate	Moderate	Limited information on the sampling strategy and data analysis
(Pechlaner et al., 2021) [77]	4/5	80%	High	Low	Adequate reporting, but limited generalizability
(Manzoor and Wei, 2018) [78]	2/5	40%	Low	High	Significant methodological issues and lack of transparency
(T Li et al., 2020) [79]	4/5	80%	High	Low	Well-conducted study, but limited discussion of limitations
(Xu et al., 2021) [63]	5/5	100%	High	Low	Well-conducted study with clear reporting and robust methodology
(Himaz, 2021) [80]	3/5	60%	Moderate	Moderate	Limited information on the data collection process and potential confounders
(Colak and Lu, 2022) [81]	4/5	80%	High	Low	Adequate reporting, but limited discussion of limitations
(Hameed et al., 2020) [82]	3/5	60%	Moderate	Moderate	Limited information on the sampling strategy and data analysis
(Ahmad and Ullah, 2023) [32]	4/5	80%	High	Low	Well-conducted study, but small sample size
(Xu, 2019) [83]	3/5	60%	Moderate	Moderate	Limited information on the data collection process and potential bias
(Liu et al., 2022) [84]	5/5	100%	High	Low	Well-conducted study with clear reporting and robust methodology
(Long and Xu, 2017) [85]	3/5	60%	Moderate	Moderate	Limited information on the sampling strategy and data analysis
(J Li et al., 2020) [86]	4/5	80%	High	Low	Adequate reporting, but limited generalizability
(Liu and Suk, 2022) [87]	4/5	80%	High	Low	Well-conducted study, but limited discussion of limitations
(Koh and Kwok, 2017) [46]	2/5	40%	Low	High	Significant methodological issues and lack of transparency
(Zhifei and Chenchen, [19]	3/5	60%	Moderate	Moderate	Limited information on the data collection process and potential confounders
(Manhas et al., 2014) [88]	2/5	40%	Low	High	Significant methodological issues and lack of transparency
(Guo et al., 2020) [41]	4/5	80%	High	Low	Adequate reporting, but limited discussion of limitations
(Huang et al., 2020) [56]	3/5	60%	Moderate	Moderate	Limited information on the sampling strategy and data analysis
(Kulgildinova et al., 2019) [47]	4/5	80%	High	Low	Well-conducted study, but small sample size
(Üzümcü and Alyakut, 2022) [89]	4/5	80%	High	Low	Adequate reporting, but limited generalizability
(Lazanyuk and Revinova, 2020) [90]	3/5	60%	Moderate	Moderate	Limited information on the data collection process and potential bias
(Zulfaqar et al., 2023) [91]	5/5	100%	High	Low	Well-conducted study with clear reporting and robust methodology
(Ahmad et al., 2018) [92]	3/5	60%	Moderate	Moderate	Limited information on the sampling strategy and data analysis
(Rauf et al., 2021) [93]	4/5	80%	High	Low	Adequate reporting, but limited discussion of limitations
(Wang et al., 2023) [94]	5/5	100%	High	Low	Well-conducted study with clear reporting and robust methodology
(Yao et al., 2021) [38]	4/5	80%	High	Low	Adequate reporting, but limited generalizability
(Gong, 2020) [95]	3/5	60%	Moderate	Moderate	Limited information on the data collection process and potential confounders
(Yu et al., 2023) [96]	5/5	100%	High	Low	Well-conducted study with clear reporting and robust methodology
(Kostopoulou et al., 2021) [15]	4/5	80%	High	Low	Adequate reporting, but limited discussion of limitations
(Wang, 2019) [97]	3/5	60%	Moderate	Moderate	Limited information on the sampling strategy and data analysis
(Schuhbert et al., 2020) [98]	4/5	80%	High	Low	Well-conducted study, but small sample size
(Wang, 2021) [99]	3/5	60%	Moderate	Moderate	Limited information on the data collection process and potential bias
(Mytaftsi and Tsironis, 2023) [100]	4/5	80%	High	Low	Adequate reporting, but limited generalizability
(Yang, 2020) [24]	3/5	60%	Moderate	Moderate	Limited information on the sampling strategy and data analysis
(Su et al., 2020) [101]	5/5	100%	High	Low	Well-conducted study with clear reporting and robust methodology
(Lostal and Vasconcelos Vilaça, 2015) [102]	2/5	40%	Low	High	Significant methodological issues and lack of transparency
(Winter, 2021) [14]	4/5	80%	High	Low	Adequate reporting, but limited discussion of limitations
(Vasconcelos Vilaça, 2018) [103]	3/5	60%	Moderate	Moderate	Limited information on the data collection process and potential confounders
(Knutson, 2020) [104]	4/5	80%	High	Low	Well-conducted study, but small sample size
(Franklin, 2023) [57]	5/5	100%	High	Low	Well-conducted study with clear reporting and robust methodology

Table 1 above presents a comprehensive assessment of the methodological quality of the studies included in this systematic review, evaluated using the Mixed Methods Appraisal Tool (MMAT), version 2018 [65]. The results reveal a wide range of methodological rigor among the included studies, with quality scores ranging from 2/5 (low quality) to 5/5 (high quality). Many studies fall within the moderate (3/5) and high (4/5) quality categories.

This distribution of scores highlights the varying levels of methodological soundness among the included studies, as determined by the rigorous MMAT criteria. In constructing Table 1, each study was evaluated against the MMAT criteria, with the results presented as both the number of criteria met (out of 5), and an overall quality score (high, moderate, or low) based on this. The order of studies in Table 1 mirrors their appearance in the subsequent Table 2, ensuring a coherent and navigable reference for readers.

**Table 2:** The 2 domains and 10 sub-domains covered in the tourism and heritage literature on the BRI. Source: edited by the authors.

Domains	Sub-domains	Themes	Geography	Authors
Tourism	Tourist flows and destinations	Global tourist flows under the BRI	Global	(Shymanskyi et al., 2022) [20]
		Modeling the flow of Chinese tourists to the "Silk Road"	Global	(Deng and Hu, 2019) [26]
		The BRI’s effect on tourism demand in China	China, Russia and Mongolia	(Li et al., 2021) [66]
		The BRI and tourist flows	Russia	(Kuchumov and Testina, 2020) [67]
		Destination attractiveness in Silk Road tourism in Uzbekistan	Uzbekistan	(Raimkulov et al., 2021) [68]
		Enhancing Silk Road Tourism via Geoinformation Technologies	Global	(Tikunov et al., 2017) [69]
		U.S. Visitor Loyalty and Heritage Tourism in Uzbekistan’s Silk Road	Uzbekistan	(Juraturgunov et al., 2023) [70]
		China- Pakistan Economic Corridor (CPEC): Boosting Connectivity and Tourism Potential	Pakistan	(Ali et al., 2017) [71]
	Tourism development	The New Silk Road and tourism development in Slovakia	Slovakia	(Kučerová et al., 2020) [72]
		International tourism development in the BRI	Global	(J Chen et al., 2021) [73]
		The BRI and inbound tourism development in Uyghur region	China and Central Asia	(Idikut, 2020) [74]
		Tourism development potential in the Chinese’s BRI provinces	China	(Y Chen et al., 2021) [45]
		Tourism development through the BRI and residents in Urumqi, Xinjiang	China	(Chan et al., 2018) [16]
		The BRI and the local stakeholders’ views on the prospects of tourism development in Kazakhstan	Kazakhstan	(Daye et al., 2020) [75]
		Spatial tourism development in the Yellow River Basin in China	China	(Zhang et al., 2020) [76]
		New Silk Road infrastructure and developing a tourism environment for residents’ life quality	Kazakhstan	(Mamirkulova et al., 2020) [5]
		Tourism development and local service through infrastructure projects along the New Silk Road in Georgia	Georgia	(Pechlaner et al., 2021) [77]
		Boosting Pakistan’s tourism through the CPEC) infrastructure	Pakistan	(Manzoor and Wei, 2018) [78]
	The tourism economy	The BRI and boosting the tourism economy	Global	(T Li et al., 2020) [79]
		Brand equity along the Silk Road route	Global	(Xu et al., 2021) [63]
		The BRI’s challenges in recent economics literature	Global	(Himaz, 2021) [80]
		The BRI’s impact on the development of the tourism economy in the Silk Road’s Chinese regions	China	(Colak and Lu, 2022) [81]
		Tourism economy boosting through CPEC: Focusing on Gwadar	Pakistan	(Hameed et al., 2020) [82]
		The BRI’s Positive Influence on Global Tourism Dynamics	Global	(Ahmad and Ullah, 2023) [32]
	Tourism competitiveness and cooperation	Tourism competitiveness in Central Asian	Central Asia	(Xu, 2019) [83]
		Tourism competitiveness and spatial differentiation in Xinjiang, China	China	(Liu et al., 2022) [84]
		Enhance tourism industry competitiveness in Hubei province under OBOR	China	(Long and Xu, 2017) [85]
		Competition and tourism cooperation along the "Silk Road Economic Belt"	Russia	(J Li et al., 2020) [86]
		China’s tourism development strategy under the OBOR in Azerbaijan	Azerbaijan	(Liu and Suk, 2022) [87]
		Rediscovering the Silk Road and regional integration in Central Asia	Central Asia	(Koh and Kwok, 2017) [46]
		International tourism cooperation based on the BRI	Global	(Zhifei and Chenchen, [19]
		The Silk Road and tourism internationalization in light of circuit tourism	Global	(Manhas et al., 2014) [88]
	Investment and marketing	Potential spillover effects of BRI on Chinese tourism to Australia	Australia and Southeast Asia	(Guo et al., 2020) [41]
		The BRI and stimulating China’s inbound tourism market	China	(Huang et al., 2020) [56]
		Problems in developing the tourism industry along Kazakh sections of the Silk Routes	Kazakhstan	(Kulgildinova et al., 2019) [47]
		Digital Revival of Silk Road Tourism	Global	(Üzümcü and Alyakut, 2022) [89]
		Digital Silk Road: Technological Transformation in Eurasia	Russia	(Lazanyuk and Revinova, 2020) [90]
		Enhancing Gilgit Baltistan Tourism through CPEC Development	Pakistan	(Zulfaqar et al., 2023) [91]
	Sustainable tourism and the environment	Tourism and Pollution in Western China’s OBOR Provinces	China	(Ahmad et al., 2018) [92]
		Transportation, energy consumption, tourism development, and environmental degradation	China	(Rauf et al., 2021) [93]
		Sustainability Dynamics in BRI Tourism and Economic Corridors	Global	(Wang et al., 2023) [94]
Heritage	Heritage protection and preservation	Proportionate distributions in the spatiotemporal structure of World Cultural Heritage Sites	Global	(Yao et al., 2021) [38]
		The intangible cultural heritage and its productive protection under the BRI	China	(Gong, 2020) [95]
		The adaptive evolution of cultural ecosystems and cultural tourism heritage along the Silk Road in China	China	(Yu et al., 2023) [96]
	Cultural tourism routes	Polycentric tourism development and Silk Road Heritage branding	Macedonia and Greece	(Kostopoulou et al., 2021) [15]
		Relational heritage sovereignty and the Silk Roads	Global	(Wang, 2019) [97]
		Cultural tourism routes as incubators for economic diversification and innovation of the BRI in Azerbaijan	Azerbaijan	(Schuhbert et al., 2020) [98]
		Ethnic minorities and the construction of sports and heritage corridors in the context of OBOR	China and Vietnam	(Wang, 2021) [99]
		Thessaloniki’s Dark Tourism: Connecting Silk Road History	Greece	(Mytaftsi and Tsironis, 2023) [100]
	Cultural heritage and tourism development	China’s urban development and producing imaginations of the Silk Road in Xi’an	China	(Yang, 2020) [24]
		Relational authenticity and reconstructed heritage at Silk Road Dingding Gate	China	(Su et al., 2020) [101]
	Challenges and opportunities	Opportunities and challenges for China from the Bamiyazation phenomenon of cultural heritage along the BRI	Global	(Lostal and Vasconcelos Vilaça, 2015) [102]
		The Silk Roads and their geocultural heritage	Global	(Winter, 2021) [14]
		Ancient Chinese thought, and strengthening the cultural normative foundations of the BRI	Global	(Vasconcelos Vilaça, 2018) [103]
		The Silk Road and archaeology	Eastern Europe	(Knutson, 2020) [104]
		The Silk Road’s Archaeology: Challenges of Storytelling and Scale	Global	(Franklin, 2023) [57]

Furthermore, the MMAT Score (%) column represents the percentage of criteria met by each study, calculated as (MMAT criteria met / 5) \* 100. The Risk of Bias column assesses the potential bias in each study based on the information provided in the manuscript and the comprehensive MMAT evaluation. Finally, the Notes column offers concise observations on the strengths, limitations, or specific issues identified for each study, providing a more detailed understanding of the methodological landscape of the included research.

### 3.6 Data synthesis

A bibliometric analysis was conducted using Litmaps software to visualize the interconnections between the included studies (S4 Fig). Studies were also classified by publisher, journal, date, author, geography, and research area (Tables 4, 5 and S5 Fig). A narrative synthesis was then performed to summarize the findings, with studies grouped according to their focus on tourism or heritage, and key themes identified.

## 4 Results

### 4.1 Overview of selected articles

The systematic literature review identified 56 relevant articles published between 2013 and 2023, with a notable increase in publications after 2017. Inductive analysis revealed two main domains (Tourism and Heritage) and ten sub-domains (Table 2) within the literature on the BRI’s impact on tourism and heritage along the Silk Roads.

### 4.2 Tourism domain

The tourism domain encompassed six sub-domains: tourist flows and destinations, tourism development, tourism economy, tourism competitiveness and cooperation, investment and marketing, and sustainable tourism and environment.

#### 4.2.1 Tourist flows and destinations

Shymanskyi et al. (2022) identified distance as a mobility barrier, with shared religions and languages having the opposite effect [20]. Deng and Hu (2019) revealed that cultural/geographic proximity between Chinese outbound markets spurs visitation, suggesting that costs and familiarity are determinants [26]. Li et al. (2021) showed that transport links, infrastructure, GDP, and internet penetration influence arrival volumes [66]. Kuchumov and Testina (2020) connected BRI frameworks with upgraded tourism infrastructure across member states [67]. Raimkulov et al. (2021) highlighted cultural allure, hospitality, and amenities as crucial for destination loyalty [68]. Tikunov et al. (2017) advocated integrated, multilingual portals to showcase dispersed Silk Road attractions [69]. Juraturgunov et al. (2023) revealed that extended stays and heritage engagement augment traveler attachments [70]. Ali et al. (2017) discussed prerequisites around security and infrastructure to unlock China-Pakistan tourism amidst BRI cooperation [71].

Various factors influencing international tourism in the context of the BRI have been analyzed, including visa regulations, culture and linguistic, transportation connectivity and coordinated marketing efforts. However, the focus has been imbalanced, with a concentration on Chinese outbound flows.

#### 4.2.2 Tourism development

Kučerová et al. (2020) found that geopolitical tensions around legal frameworks have hindered tourism progress in Slovakia despite BRI cooperation efforts. They emphasized the need for strategic collaboration among stakeholders with complex interests [72]. Chen et al. (2021) identified positive linkages between BRI and tourism revenue expansion, leading to regional economic development. However, they highlighted the importance of governance mechanisms to ensure equitable growth sharing [73]. Idikut (2020) focused on tourism promotion prerequisites in Xinjiang, emphasizing the balance between security priorities and tourism growth [74]. Chen et al. (2021) proposed a multidimensional assessment framework to quantify tourism development potentials and planning needs [45]. Chan et al. (2018) emphasized the importance of attuning balanced enhancement approaches with cultural preservation based on local community inputs in Xinjiang [16]. Daye et al. (2020) found overall stakeholder support in Kazakhstan for BRI tourism infrastructure advancement, but concerns remained regarding unmoderated external cultural assimilation pressures [75]. Zhang et al. (2020) demonstrated significant infrastructure investment effects on stimulating regional tourism economies, as evidenced in China’s Yellow River Basin [76]. Mamirkulova et al. (2020) evaluated the New Silk Road Initiative outcomes on living standards in Kazakhstan spanning social, economic, ecological, and cultural dimensions [5]. Pechlaner et al. (2021) highlighted the need for cooperation policies adapted to members’ development levels alongside fostering local enterprise participation for sustainable tourism growth [77]. Manzoor & Wei (2018) projected that the CPEC blueprint could improve Pakistan’s tourism competitiveness by expanding connectivity channels, heightening investments and easing access to natural heritage sites, provided security prerequisites are established [78].

These studies showcase the importance of governance collaboration, public-private partnerships, and community participation models in tourism development, as well as the need to balance conservation and development.

#### 4.2.3 Tourism economy

Through panel analyses, Li et al. (2020) demonstrated that the BRI has expanded inbound tourist volumes and receipts. As a result, they recommend differentiated management, deeper cooperation, and improved connectivity to further boost positive effects on the tourism economy [79]. Xu et al. (2021) formulated a road-trip tourism brand equity model to inform marketing strategies around heightening loyalty and engagement [63]. Himaz (2021) flagged risks like unbalanced investments, escalating debt, and inequality that can accompany tourism-centered growth, underscoring sustainable, ethical practices [80]. Colak & Lu (2022) revealed significant improvements in per capita tourism indicators in China under BRI programs, confirming positive contributions and necessitating coordinated policy oversight [81]. Hameed et al. (2020) employed computational modeling to reveal factors determining tourism destination competitiveness. They highlighted infrastructure development and promotion essentials for transforming the coastal Pakistani town of Gwadar into an international attraction [82]. Ahmad & Ullah (2023) uncovered increased inbound travel and revenues from a panel analysis of 140 BRI member states, with pronounced tourism economy gains in South/Western Asian regions. They have also advocated for more cooperation and private-sector strategies to leverage these trends [32].

The literature has connected the BRI to positive outcomes like rising visitor numbers and revenue. Criticism has also emerged regarding financial sustainability, uneven benefits distribution, and reliance on Chinese travelers.

#### 4.2.4 Tourism competitiveness and cooperation

Xu (2019) has advocated for government efforts in devising tourism plans, further enabling projects, and harnessing cultural heritage to boost industry growth [83]. Liu et al. (2022) investigated geographical variances in competitiveness metrics across Chinese provinces attributable to differential resource allocations, infrastructure, and endowments [84]. Long & Xu (2017) proposed calibrated policy coordination models between local territories to enhance collective competitiveness [85]. Li et al. (2020) examined collaboration requirements in Northwestern China to create integrated tourism development systems [86]. Liu & Suk (2022) recommended sustainable practices and increased bilateral partnerships between Azerbaijan and China to achieve balanced tourism progression [87]. Koh & Kwok (2017) emphasized the importance of marketing, connectivity, and governance in unlocking Central Asia’s tourism potential within cooperative BRI frameworks despite political tension [46]. Zhifei & Chenchen (2020) highlighted the challenges of nascent tourism industries but also the substantial potential for improving competitive tourism sectors through better cultural products, coordinated systems, and infrastructure modernization, fostering regional integration [19]. Manhas et al. (2014) emphasized cooperative imperatives around co-branding, community participation, and environmental audits for optimizing tourism growth across Silk Road territories [88].

Taken together, these studies indicate that the tourism industry could be more competitive by improving infrastructure, coordinating regional efforts, and utilizing cultural assets. However, at present, political tension is posing barriers to achieving these goals.

#### 4.2.5 Investment and marketing

Guo et al. (2020) have identified several factors that determine the entry of Chinese investors into Australia, such as diplomatic ties, aviation links, and visa policies. Furthermore, conditions for tourism marketing systems should be established to take advantage of Australia’s proximity to major Asian markets [41]. Huang et al. (2020) emphasized the importance of developing distinctive marketing strategies for China that consider the economic and developmental profiles of the source countries [56]. Kulgildinova et al. (2019) highlight Kazakhstan’s underleveraged cultural tourism promise, advocating prioritized domestic build-up before targeting international travelers through coordinated promotion and state-supported campaigns [47]. Üzümcü & Alyakut (2022) make the case for digitally reviving Silk Road tourism through virtual cultural dissemination technologies as avenues for heritage preservation and destination marketing [89]. Lazanyuk & Revinova (2020) spotlight the potential of harnessing technologies like AI, Big Data, and Blockchain for reimagining logistics, commerce, and tourism across the Eurasian Silk Road region. However, contextual limitations around preparative policy frameworks prevail [90]. Zulfaqar et al. (2023) quantitatively verified positive linkages between infrastructure expansion and regenerative tourism growth in Pakistan, underscoring calibrated planning for optimization [91].

The existing research has linked digitization, coordinated campaigns, and tourism data systems to attracting investment and showcasing Silk Road heritage. However, implementation remains fragmented as of this writing. Prioritizing domestic travelers where appropriate is one solution which has been suggested.

#### 4.2.6 Sustainable tourism and the environment

Ahmad et al. (2018) have studied the ecological damage caused by tourism across different provinces of China associated with projects under the BRI. They highlight the importance of regulatory and energy measures to balance growth and sustainability [92]. Rauf et al. (2021) have found positive connections between transportation, hotels and emissions. They suggest green investment to reconcile these factors by relaxing international tourist visa rules [93]. Wang et al. (2023) have employed econometric analysis to show that pollution initially increases with development, but eventually plateaus due to the adoption of cleaner technologies that are the result of government policies. All these studies point towards the need for nuanced frameworks to integrate economic priorities and environmental dimensions within tourism expansion under BRI auspices [94].

While studies show that tourism expansion and transport development pose environmental threats, there is currently a lack of investigations examining locally relevant sustainable tourism models.

### 4.3 Heritage domain

The heritage domain included four sub-domains: heritage protection and preservation, cultural tourism routes, cultural heritage and urban development, and challenges and opportunities.

#### 4.3.1 Heritage protection and preservation

Yao et al. (2021) studied the preservation requirements of cultural heritage sites along the Silk Roads. They examined these sites’ geospatial distribution dynamics and developmental, locational, and typological attributes to reveal the necessary preservation measures [38]. Gong (2020) explored the mechanisms that promote living inheritance systems for intangible cultural heritage. The study suggests that elevating the economic valuation and opportunities associated with the heritage can help sustain it [95]. Yu et al. (2023) propose adaptive heritage governance regimes that balance development priorities with conservation needs, limiting tourism in fragile areas, while promoting regulated access elsewhere [96].

The discussions on heritage preservation emphasize the importance of digitization and controlled development. This approach simultaneously enables poverty alleviation, human capital development, and heritage preservation. Experts call for nuanced cultural integration rather than tourism-centric approaches across Silk Road countries intersecting with BRI connectivity and developmental blueprints.

#### 4.3.2 Cultural tourism routes

Kostopoulou et al. (2021) have investigated how combining branding and experience development can help boost economic growth in less-developed regions along the Silk Road by reviving heritage [15]. Wang (2019) looked into the geopolitical dimensions of constructing heritage sites externally versus interpreting their meaning and identity internally [97]. Schuhbert et al. (2020) formulated a competitive strategy that leverages cultural tourism to promote economic diversification by identifying regional clusters and local needs in Azerbaijan [98]. Wang (2021) examined the requirements for developing heritage corridors while expanding railway infrastructure projects to preserve minority community practices equitably [99]. Mytaftsi & Tsironis (2023) explored integrating dark historical events with spiritual traditions to enhance multifaceted cultural tourism representations. The findings have revealed both benefits and drawbacks of heritage tourism development under connectivity schemes like the BRI [100].

The literature has identified the economic, social, and political benefits of reviving ancient Silk Road trails as contemporary tourism pathways. However, the main challenges relate to the commercialization of heritage sites and lack of participation from minority populations.

#### 4.3.3 Cultural heritage and urban development

Yang (2020) shed light on the governance tensions between the BRI’s top-down policies and the bottom-up grassroots interpretations of the Silk Road by various stakeholders during the localization process. The findings suggest that it is important to consider the ethnic, religious, and linguistic diversity when developing cultural strategies [24]. In a separate study, Su et al. (2020) investigated the challenge of preserving heritage authenticity in rapidly changing urban environments while also promoting tourism. They looked at the Luoyang Silk Road Dingding Gate project, which highlights the need for a balance between preservation, renewal, and tourism [101].

Both studies suggest the potential to use the Silk Road’s historical affiliations to drive urban renewal and tourism growth. However, there may be tension between different communities’ interpretations of cultural symbolism, which could complicate these efforts.

#### 4.3.4 Challenges and opportunities

Lostal & Vasconcelos Vilaça (2015) wrote about the threat of "Bamiyazation," which refers to the intentional destruction of cultural heritage. They proposed that China implement comprehensive protective measures, including introducing "crimes against common cultural heritage." They also suggested aligning the Belt and Road Initiative (BRI) with international cultural policies to enhance China’s global influence [102]. Winter (2021) discussed how Silk Road tourism is increasingly seen as a cultural metaphor for promoting friendship, but it requires consistent policy implementation to be effective [14]. Vasconcelos Vilaça (2018) addressed accusations of colonial posturing by emphasizing that representation should be created through reciprocal participation [103]. Knutson (2020) argued that Silk Road tourism can be used as an analytical tool to understand the interactions between global phenomena and local cultures [104]. Franklin (2023) used allegory to draw attention to the tension involved in positioning the Silk Roads as both universally appealing attractions and culturally specific places. The findings underscore the need for equitable promotion of tourism while preserving heritage diversity [57].

These studies suggest several solutions, including implementing international policies for heritage protection, embracing the diverse history of the Silk Road through reciprocal participation, and using nuanced analytical frameworks to integrate its various dimensions.

### 4.4 Interconnections between themes

A bibliometric analysis using Litmaps software (S4 Fig) revealed interconnections between themes, such as the relationship between tourism development and tourist flows/destinations, the interrelation between tourism industry and investment/marketing strategies, and the convergence of tourism, economic development, and cultural heritage preservation within the BRI context.

### 4.5 SWOT analysis

A SWOT analysis (Table 3) highlighted the strengths, weaknesses, opportunities, and threats related to the emerging research discourse on the BRI’s influence on tourism and cultural heritage along the Silk Roads.

**Table 3:** SWOT analysis based on the review’s results. Source: edited by the authors.

**Strengths +**	**Weaknesses −**
Momentum of BRI construction expanding regional transport connectivity and catalyzing infrastructure growth High tourist interest and investment in Silk Road cultural heritage Existing institutional frameworks around conservation and development Cultural capital concentrated across interconnected Silk Road locations Tourism expansion and revenue-generation prospects Rising global awareness and demand	Challenges in comprehensively estimating the impacts of the BRI’s global tourism and heritage due to insufficient articles, and a lack of linguistic diversity and long-term data Imbalances in research attention skewed towards certain regions/themes and concentrated selectively Underdeveloped cultural tourism infrastructure in remote Silk Road outposts Limited policies upholding sustainability, equity, social welfare, and cultural autonomy Constraints around inter-governmental coordination complexity
**Opportunities +**	**Threats −**
Harnessing heritage sites simultaneously for preservation, poverty alleviation, and ownership Regional coordination to ease cross-border tourist flows, infrastructure upgrades, and diffusion of prosperity Reviving cultural routes to spur economic growth across communities and stimulate local enterprise and employment Harnessing heritage sites for preservation, poverty alleviation and ownership Further illuminating overlooked regions such as the Middle East, which possess rich history and cultural ties to the Silk Roads	Uncontrolled tourism and transport expansion damaging ecological habitats Cultural commodification morphing rich traditions into superficial attractions Uneven economic accruement concentrating gains across nodes and exacerbating leaks Economic leakage consolidating gains unevenly across nodes Social tensions around rights, identity, and representation

### 4.6 Sensitivity analysis

We conducted sensitivity analyses to explore the robustness of the review findings to study quality. The analysis involves repeating the qualitative synthesis, including only high-quality studies (i.e., those meeting all or most of the MMAT criteria) [65].

The sensitivity analysis revealed that the overall conclusions of the review remained largely unchanged when considering only high-quality studies. The main themes and patterns identified in the original analysis, such as the focus on economic and infrastructural aspects of tourism, the need for sustainable tourism models, and the importance of cultural heritage preservation, were still evident in the high-quality studies.

However, some nuance and additional insight emerge from sources other than the sensitivity analysis. For example, the high-quality studies provided more detailed and reliable data on the specific effects of the BRI on tourism flows, investment, and local communities. They also offered more robust evidence for the effectiveness of certain strategies, such as community engagement and international cooperation, in promoting sustainable tourism and heritage conservation.

The sensitivity analyses based on study quality confirm the main findings of the systematic review while highlighting the importance of methodologically rigorous research in understanding the complex relationships between the BRI, tourism, and cultural heritage along the Silk Roads.

### 4.7 Summary

The analysis of the emerging research reveals a complex, multifaceted discourse centered on 10 pivotal themes. The findings show substantial potential for tourist flows, destination development, urban renewal, heritage preservation, and revived cultural routes if integrated policies, public-private collaboration, and equitable community participation frameworks are implemented with care and attention to ecological limits. However, acute challenges around financial imbalances, uneven access, social disruption, cultural commodification, and environmental damage could arise without careful, context-specific planning attentive to sustainability.

## 5 Discussion

### 5.1 General findings

This systematic literature review has examined the BRI’s impact on tourism and heritage along the Silk Roads. Though research on tourism and cultural heritage under the BRI has been scant, several key findings and observations can be drawn from the analysis (Tables 4, 5 and S5 Fig).

**Table 4:** Classification of the 56 selected studies by publisher and journal. Source: edited by the authors.

Publisher	Number of Studies	Percentage	Journal	Number of Studies	Percentage
MDPI	11	20%	Sustainability	10	~ 18%
			Land	1	~ 2%
Taylor & Francis (Routledge)	12	~ 22%	Current Issues in Tourism	1	~ 2%
			Service Industries Journal	3	~ 6%
			Asia Pacific Journal of Tourism Research	1	~ 2%
			Territory, Politics, Governance	1	~ 2%
			International Journal of Cultural Policy	1	~ 2%
			International Journal of Heritage Studies	1	~ 2%
			World Archaeology	1	~ 2%
			Journal of China Tourism Research	2	~ 4%
			Journal of Quality Assurance in Hospitality and Tourism	1	~ 2%
Elsevier	4	~ 7%	Journal of Destination Marketing and Management	1	~ 2%
			Tourism Management Perspectives	1	~ 2%
			Global Ecology and Conservation	1	~ 2%
			Procedia–Social and Behavioral Sciences	1	~ 2%
SAGE	3	~ 6%	Tourism Economics	2	~ 4%
			Evaluation Review	1	~ 2%
Public Library of Science	3	~ 6%	PLOS ONE	3	~ 6%
Springer	5	~ 9%	Journal of Archaeological Research	1	~ 2%
			China and the New Silk Road	2	~ 4%
			Normative Readings of the Belt and Road Initiative	1	~ 2%
			Cities’ Vocabularies and the Sustainable Development of the Silkroads	1	~ 2%
Walter de Gruyter GmbH	1	~ 2%	Zeitschrift fur Wirtschaftsgeographie	1	~ 2%
Lomonosov Moscow State University, Faculty of Geography	1	~ 2%	Geography, Environment, Sustainability	1	~ 2%
Hindawi	1	~ 2%	Discrete Dynamics in Nature and Society	1	~ 2%
Oxford University Press	1		Chinese Journal of Comparative Law	1	~ 2%
The Canadian Center of Science and Education (CCSE)	2	~ 2%	International Business Research	1	~ 2%
			Journal of Management and Sustainability	1	~ 2%
Sciendo fa parte della società De Gruyter	1	~ 2%	Confrontation and Cooperation: 1000 Years of Polish-German-Russian Relations	1	~ 2%
Scientific Research Publishing Inc	1	~ 2%	Advances in Applied Sociology	1	~ 2%
Atlantis Press	7	~ 13%	Advances in Economics, Business and Management Research	3	~ 6%
			Advances in Social Science, Education and Humanities Research	4	7%
World Center of Innovation Research and Publication	1	~ 2%	New Trends and Issues Proceedings on Humanities and Social Sciences	1	~ 2%
IOP Science	1	~ 2%	IOP Conference Series: Earth and Environmental Science	1	~ 2%
IGI Global	1	~ 2%	Normative Readings of the Belt and Road Initiative	1	~ 2%

**Table 5:** Classification of the 56 selected studies by date, first author’s nationality, geography and research area. Source: edited by the authors.

Date	First Author’s Nationality	Geography	Research Area
2023 (7) 2022 (5) 2021 (12) 2020 (18) Total 42 papers [75%]	Chinese, 24 papers [43%]	China (13) China and Central Asia (1) China, Russia and Mongolia (1) China and Vietnam (1) Total 16 papers [29%]	Tourism Tourist flows and destinations (8) Tourism development (10) The tourism economy (6) Tourism competitiveness and cooperation (8) Investment and marketing (6) Sustainable tourism and the environment (3) Total 41 research papers [73%]
2019 (4) 2018 (4) 2017 (4) 2015 (1) 2014 (1) Total 14 papers [25%]	Other nationalities, 32 papers [57%]	Global (18) Central Asia (2) Australia and Southeast Asia (1) Russia (3) Kazakhstan (3) Uzbekistan (2) Pakistan (4) Azerbaijan (2) Georgia (1) Eastern Europe (1) Macedonia and Greece (1) Greece (1) Slovakia (1) Total 40 papers [71%]	Heritage Heritage protection and preservation (3) Cultural tourism routes (5) Cultural heritage and tourism development (2) Challenges and opportunities (5) Total 15 research papers [27%]

#### 5.1.1 Publisher, journal, and research output

The leading publishers of the studies were Taylor & Francis (Routledge) (22%) and MDPI (20%), with significant contributions from journals such as *Sustainability* (MDPI), *Service Industries Journal* (Taylor & Francis), and *PLOS ONE* (Public Library of Science). The analysis reveals an acceleration in research contributions, underscoring the growing attention from the international academic community toward examining the complex effects of interweaving antiquity with modern mobility.

#### 5.1.2 Authors, geography, and research areas

The nationality of the first authors shows a balanced distribution, with Chinese researchers authoring 43% of the papers and authors of other nationalities contributing 57%. Geographically, 29% of the papers focused on China or a combination of China and other countries, while 71% covered a broader global context or specific regions outside of China. The research areas were predominantly focused on tourism (73%), with 27% also on heritage.

The burgeoning body of research post-2020 underscores the growing global interest in these impacts. However, it reveals a discrepancy in geographical coverage and depth of analysis, with critical regions such as the Middle East receiving less attention, despite their historical and cultural importance.

### 5.2 Research gaps and implications

The current research landscape is imbalanced, with a significant focus on tourism development, overshadowing the crucial areas of cultural heritage preservation, local community welfare, and environmental sustainability. These findings suggest a pressing need for a more comprehensive and inclusive research approach which considers the BRI’s economic dimensions and social, cultural, and ecological implications.

Adopting a collaborative and multidisciplinary approach to research is essential, integrating viewpoints from local stakeholders, environmental scientists, historians, and policymakers to balance development and preservation [105]. The ultimate goal is to ensure that the BRI promotes mutual benefit, where economic gains do not come at the expense of cultural integrity, environmental protection, and/or equitable growth.

### 5.3 Implications of study quality and risk of bias

The quality assessment of the included studies using the MMAT revealed varying levels of methodological quality. While some studies met all or most of the criteria for the MMAT [65], others had limitations in their design, data collection, or analysis. These limitations may have affected the results of individual studies, and therefore, the overall conclusions of this systematic review.

Studies of low methodological quality may have introduced biasing or confounding factors that could have affected the accuracy and/or reliability of their results. For example, small sample sizes, lack of control groups, or inadequate statistical analyses may have led to overestimation or underestimation of the BRI’s impact on tourism and heritage along the Silk Roads.

To address these concerns, we conducted sensitivity analyses to explore the robustness of the review findings in terms of the studies’ quality. Sensitivity analyses included repeating the analysis only for studies of high quality (i.e., those that meet all or most of the MMAT criteria). The results of these analyses, which are discussed in the Sensitivity Analyses subsection, provide a more reliable estimate of the BRI’s impact on tourism and heritage, considering the potential effects of study quality and risk of bias.

### 5.4 Research limitations

Integrating existing academic literature with improved empirical monitoring mechanisms poses several challenges, including linguistic selectivity, temporal constraints, geopolitical instability, geographic imbalance, data format limitations, and quantitative data limitations. Scientific course correction is essential when responding to emerging scenarios, and cultural knowledge and empirical evidence can guide future research. Ultimately, this should lead to equitable and sustainable outcomes for Silk Road regions affected by the BRI.

## 6 Conclusions

The Belt and Road Initiative presents a significant opportunity to revive the Silk Roads and strengthen economic and cultural ties despite its unclear stance on tourism and heritage. This review highlights the need for a more detailed analysis of the BRI’s impact, and calls for a shift in research focus towards neglected areas. A comprehensive and interdisciplinary approach is crucial to uniting the interests of local communities, sustainable development, and heritage preservation. As research continues, it becomes increasingly clear that a comprehensive perspective is necessary, combining quantitative assessment with qualitative insight into regions’ cultural, ecological, and social characteristics. Thus, a concerted effort is needed to guide the way toward a path that honors the Silk Roads’ historical significance while promoting an environmentally responsible, inclusive future. Policy and practice should not only support connectivity, but also preserve cultural heritage, empower local communities, and be adapted to the unique environmental and societal contexts of the Silk Roads. In this way, the initiative can become a meaningful symbol of progress, bridging the gap between the ancient and the modern in a harmonious blend of development and preservation.

## 7 Future research agenda

Based on the findings of this systematic literature review and accounting for the limitations of current research, we suggest the following future research agenda:

1. Explore underrepresented sub-regions such as the Middle East.
2. Investigate sustainable tourism models that balance growth with ecological limits.
3. Harness innovation opportunities around digitization, experiential technologies, and data analytics.
4. Foster partnerships between state and non-state actors to support collaboration and inclusive growth.
5. Examine the long-term policy implications of tourism economy expansion, investment trends, visa regimes, and regional coordination.
6. Evaluate the importance of preserving cultural heritage and community participation frameworks.

The future research agenda could be further expanded by exploring specific research questions within major themes such as regional inclusivity, environmental sustainability, technological innovation, governance integration, risk mitigation, and revitalized connectivity.

Further research examining the various intersections under the BRI has the potential to promote effective utilization of infrastructure connectivity, economic priorities, environmental sustainability, and cultural pluralism, effecting positive change in a rapidly transforming landscape while celebrating the adventurous, collaborative, and progressive spirit of the Silk Road connections that have linked civilizations throughout the ages.

## 8 Practical application

This systematic literature review offers practical implications for tourism officials involved in the BRI, and discusses its impact on tourism and heritage along the Silk Roads. The findings can inform the development of policies that balance economic growth with the preservation of cultural and natural heritage sites, guide the implementation of sustainable tourism initiatives, and assist cultural heritage managers in preserving and enhancing heritage sites while accommodating increased tourism. Furthermore, tourism agencies and marketers can utilize the study’s insight to promote Silk Road tourism, foster international cooperation and collaboration, and encourage community engagement in the tourism sector. The comprehensive overview and identification of research gaps in this study are valuable resources for academic and research communities, explaining the development of educational programs and curricula related to sustainable tourism, cultural heritage management, and the BRI. By adopting a holistic approach that balances top-down development mandates with bottom-up considerations of cultural and environmental factors, stakeholders can work towards a more sustainable, inclusive, and culturally sensitive future for Silk Road tourism and heritage.

## Supporting information

S1 Fig The Ancient Silk Road map for UNESCO’s Silk Road project. Source: [49], edited by the authors.
(DOCX)

S2 Fig UNESCO World Heritage Sites along the Ancient Silk Roads. Source: [55], edited by the authors.
(DOCX)

S3 Fig Economic impact of tourism in the countries along the Ancient Silk Roads. Source: [64], edited by the authors.
(DOCX)

S4 Fig Bibliometric map created by Litmaps software: Each node (dot) represents a different academic article, and the lines between them indicate citations. Source: edited by the authors.
(DOCX)

S5 Fig Classification of 56 studies by publisher, journal, date, author, geography and research area. Source: edited by the authors.
(DOCX)

S1 Table PRISMA 2009 checklist. (DOCX)

S2 Table The journals in which the 56 selected studies were published were identified based on their standing in SCImago Journal Rank (SJR) and inclusion in the Scopus database (Elsevier) (38 articles, 68%). Source: edited by the authors.
(DOCX)

S3 Table The journals in which the 56 selected studies were published were identified based on their standing in Google Scholar (18 studies, 32%). Source: edited by the authors.
(DOCX)
