# Supplementary figures and images for: The Belt and Road Initiative’s impact on tourism and heritage along the Silk Roads: A systematic literature review and future research agenda
Source: PLoS One. 2024 Jul 18;19(7):e0306298. doi: 10.1371/journal.pone.0306298 (PMC11257252; doi:10.1371/journal.pone.0306298)

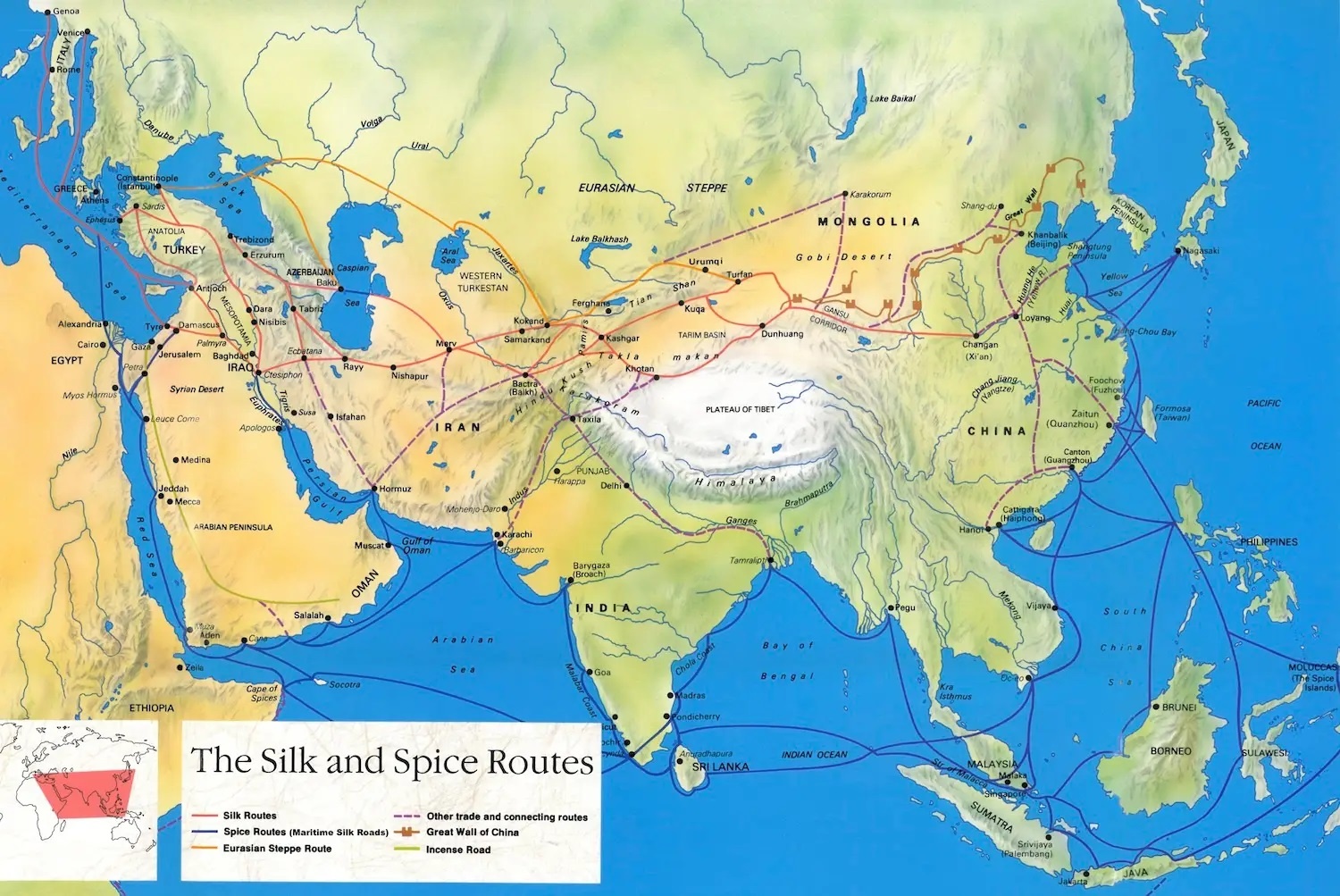


**S1 Fig．The Ancient Silk Road map for UNESCO’s Silk Road project.** Source:[49], edited by the authors

Supplement: S1 Fig — Source: [49], edited by the authors. (DOCX) [file pone.0306298.s001.docx]
