# Supplementary material for: The Belt and Road Initiative’s impact on tourism and heritage along the Silk Roads: A systematic literature review and future research agenda
Source: PLoS One. 2024 Jul 18;19(7):e0306298. doi: 10.1371/journal.pone.0306298 (PMC11257252; doi:10.1371/journal.pone.0306298)
Supplement: S2 Fig — Source: [55], edited by the authors. (DOCX) [file pone.0306298.s002.docx]

**S2 Fig. UNESCO World Heritage Sites along the Ancient Silk Roads.** Source: [55], edited by the authors
