# Supplementary material for: The Belt and Road Initiative’s impact on tourism and heritage along the Silk Roads: A systematic literature review and future research agenda
Source: PLoS One. 2024 Jul 18;19(7):e0306298. doi: 10.1371/journal.pone.0306298 (PMC11257252; doi:10.1371/journal.pone.0306298)
Supplement: S3 Fig — Source: [64], edited by the authors. (DOCX) [file pone.0306298.s003.docx]

**S3 Fig．Economic impact of tourism in the countries along the Ancient Silk Roads.** Source: [64], edited by the authors
