# Supplementary material for: The Belt and Road Initiative’s impact on tourism and heritage along the Silk Roads: A systematic literature review and future research agenda
Source: PLoS One. 2024 Jul 18;19(7):e0306298. doi: 10.1371/journal.pone.0306298 (PMC11257252; doi:10.1371/journal.pone.0306298)
Supplement: S4 Fig — Source: edited by the authors. (DOCX) [file pone.0306298.s004.docx]

***4.4 Interconnections between themes***

The bibliometric analysis using Litmaps software (S4 Fig) revealed several key interconnections between the themes identified in the literature review.

***4.4.1 Tourism Development and Tourist Flows/Destinations***

The literature on tourism development frequently explores how infrastructure and policy initiatives under the BRI can improve tourism in different regions. This naturally leads to the examination of tourist flows and the attractiveness of destinations, especially along the Silk Road. Studies have demonstrated how BRI projects impact the appeal and accessibility of tourist destinations, highlighting the close relationship between these two sub-domains [19, 23, 58].

***4.4.2 The Tourism Industry and Investment/Marketing Strategies***

Research in this field examines how investment in the BRI enhances local and regional tourism industries. Additionally, studies investigate how digital transformation, including the Digital Silk Road, modifies marketing methods and affects the tourism industry in BRI countries [38, 82, 83]. This interconnection highlights the importance of investment and marketing strategies in driving the growth and competitiveness of the tourism industry in the BRI context.

***4.4.3 Tourism Development and Environmental Sustainability***

Several research papers have examined the relationship between tourism development and environmental sustainability, emphasizing the significance of balancing economic growth and environmental conservation. These studies highlight the importance of adopting sustainable tourism practices in BRI-related developments [85–87]. This interconnection underscores the need for a holistic approach to tourism development that considers its environmental impact.

***4.4.4 Heritage Protection and Cultural Tourism Routes***

The relationship between heritage protection and the creation of cultural tourism routes is a crucial area of study. Researchers in this field aim to conserve cultural heritage while also promoting it as a part of tourism development. This involves examining methods to incorporate heritage sites into tourism routes to increase their worth and attractiveness [14, 91, 92]. This interconnection highlights the potential synergies between heritage preservation and sustainable tourism development.

***4.4.5 Cultural Heritage and Tourism Development***

This area discusses the role of cultural heritage in tourism development, exploring how the Belt and Road Initiative can preserve and showcase cultural heritage, attract tourists, and benefit local economies [21, 95]. The interconnection between these sub-domains emphasizes the importance of leveraging cultural assets for sustainable tourism growth while ensuring their preservation and authenticity.

These interconnections suggest areas for further analysis, such as exploring the impact of digital transformation on tourism marketing and the economy in BRI regions, delving deeper into sustainable tourism practices and their integration with BRI development projects, assessing the impact of BRI on local communities, investigating the role of policy in shaping tourism development and heritage conservation and exploring the impact of global events on tourism trends and destination resilience.


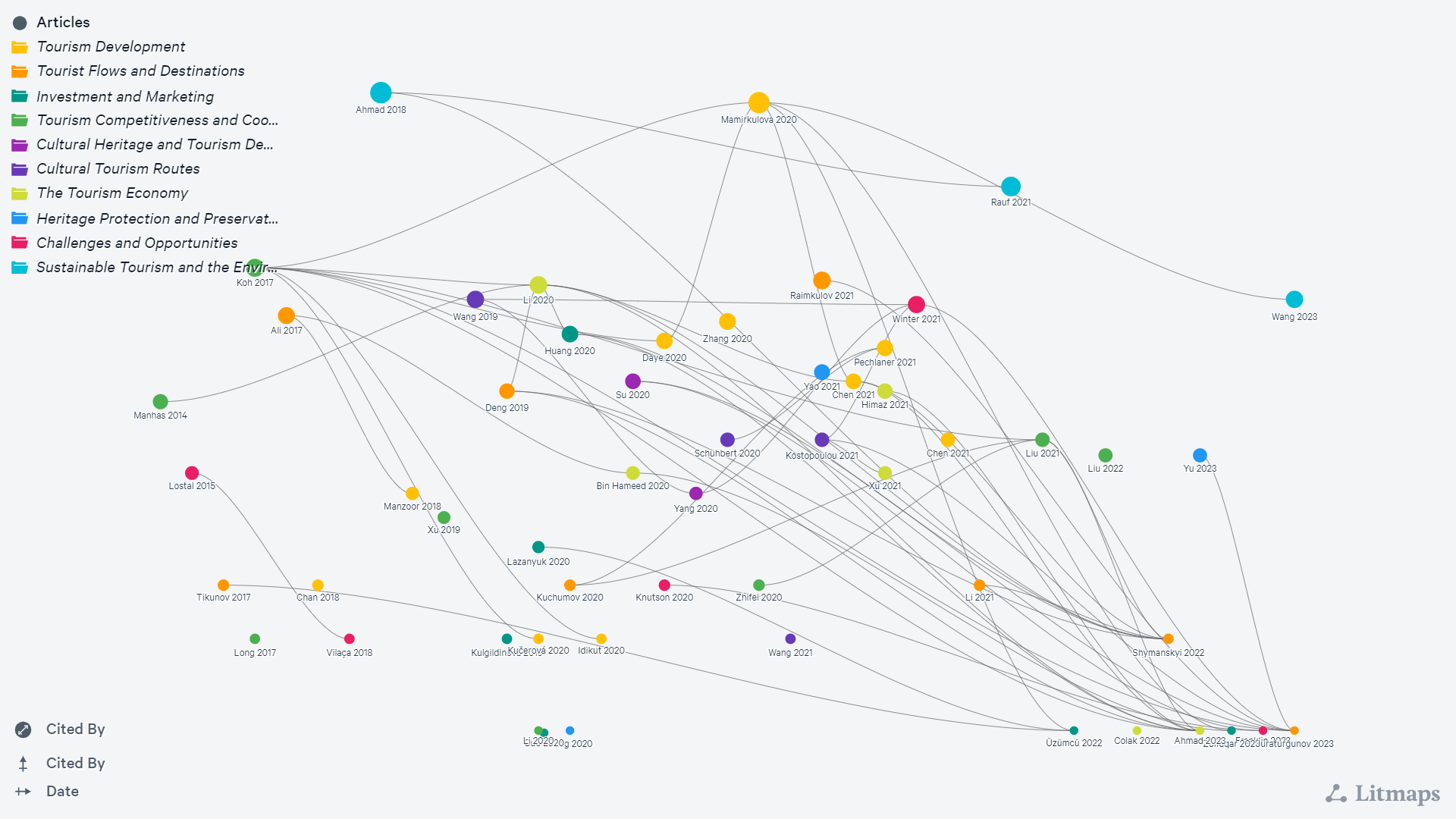


1. **Article positions, cited by (cited by & date)**


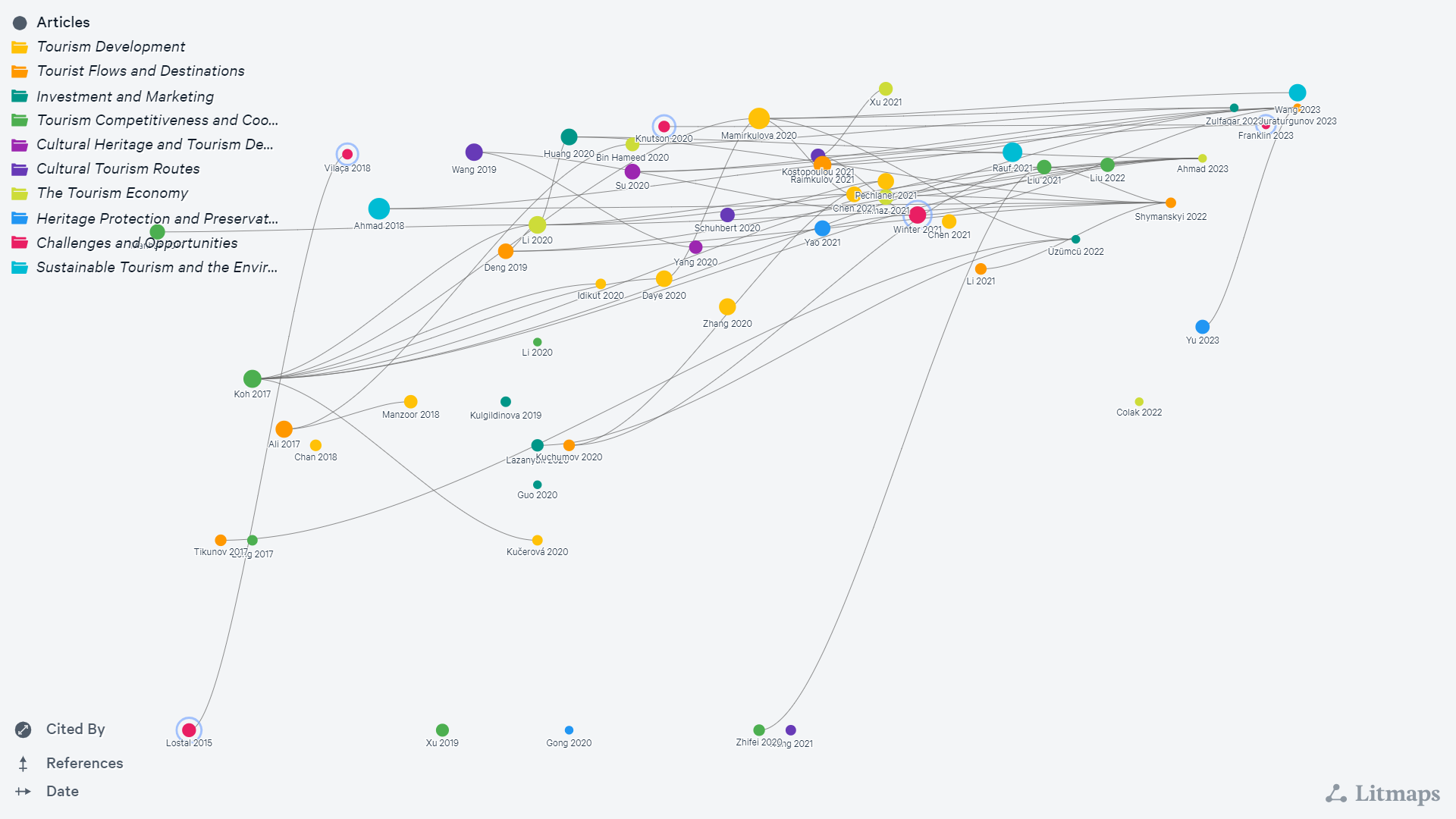


1. **Article positions, cited by (date & date)**


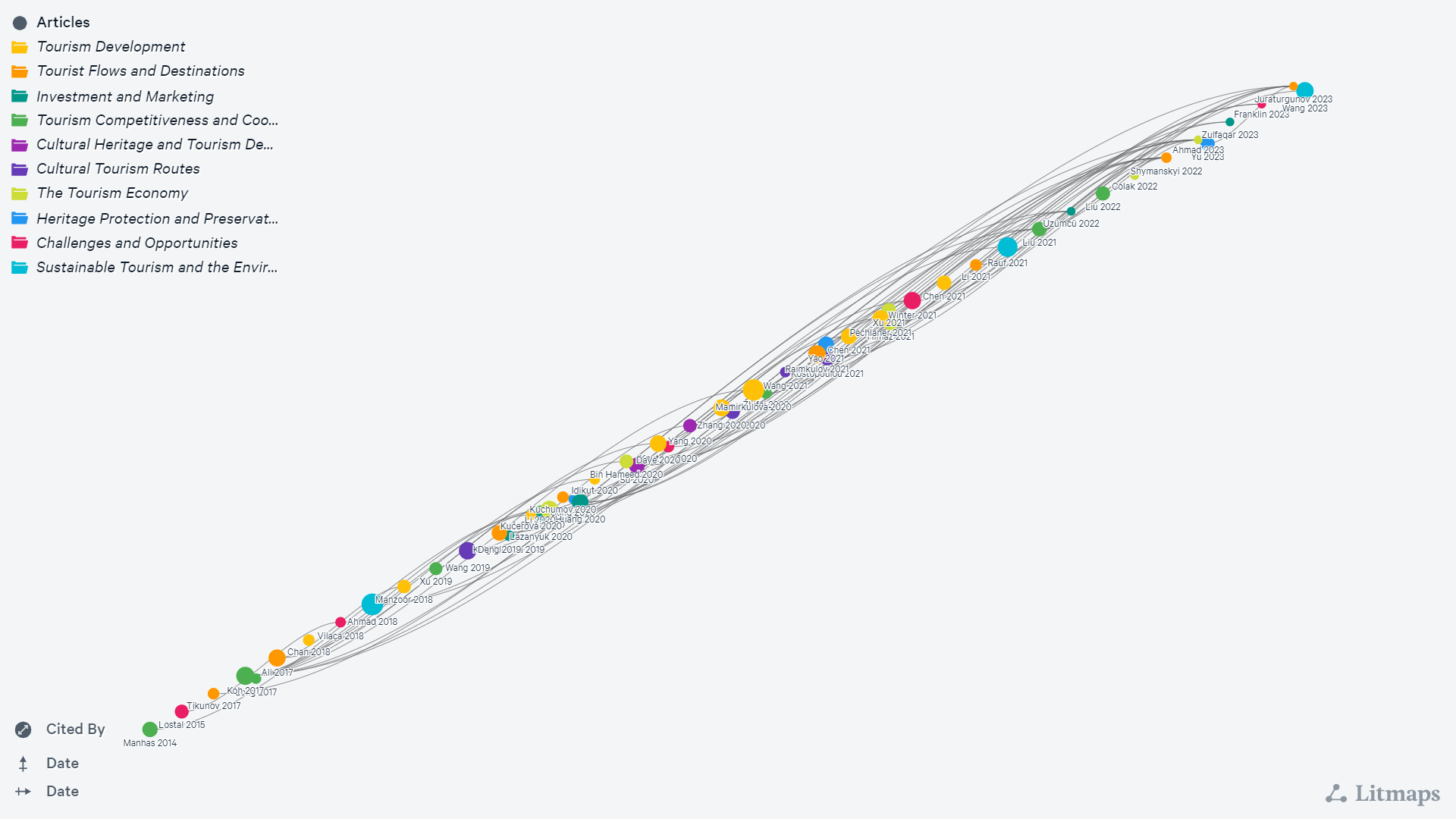


1. **Article positions, cited by (references & date)**


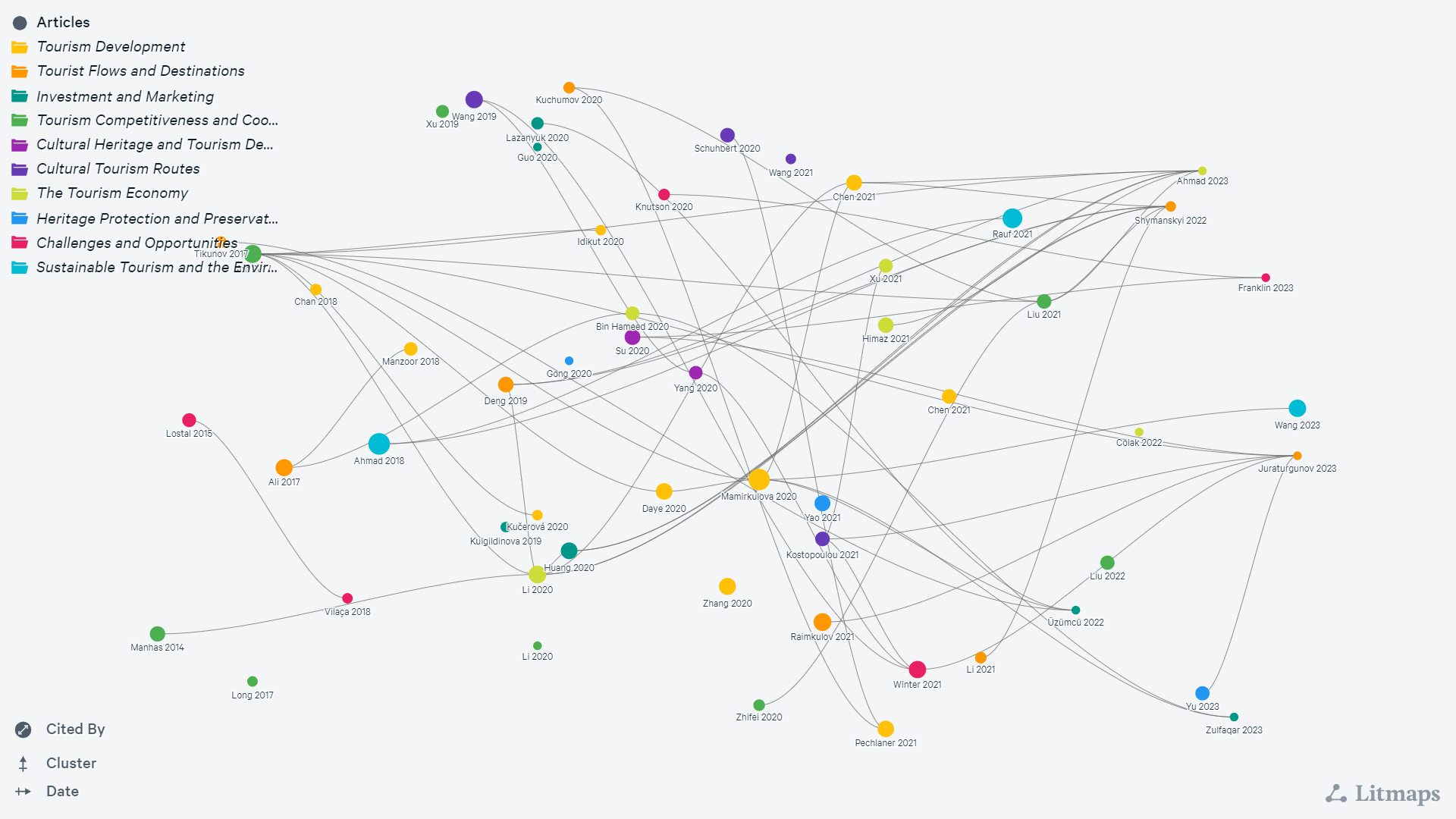


1. **Article positions, cited by (cluster & date)**

**
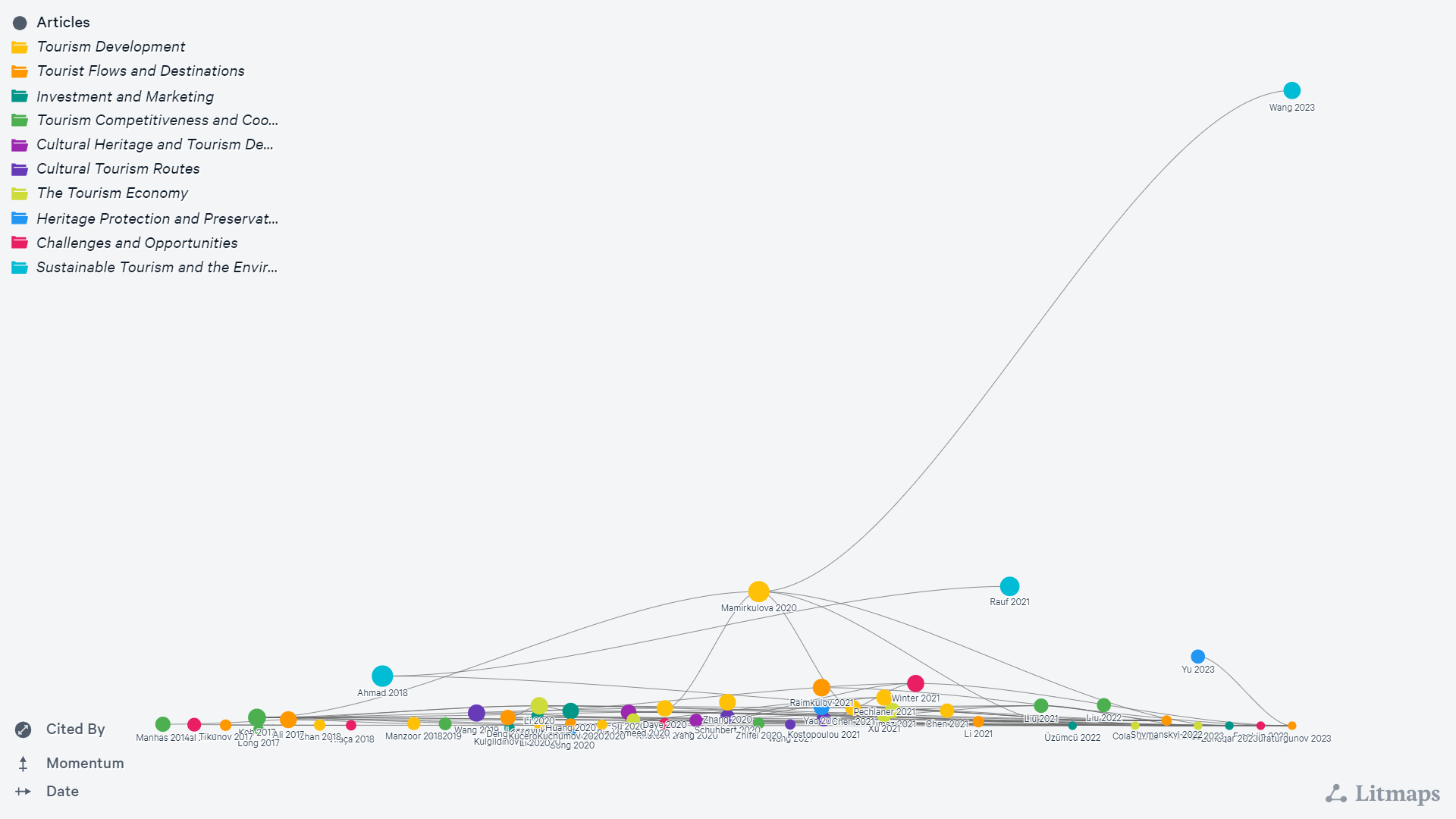
**

1. **Article positions, cited by (momentum & date)**

**
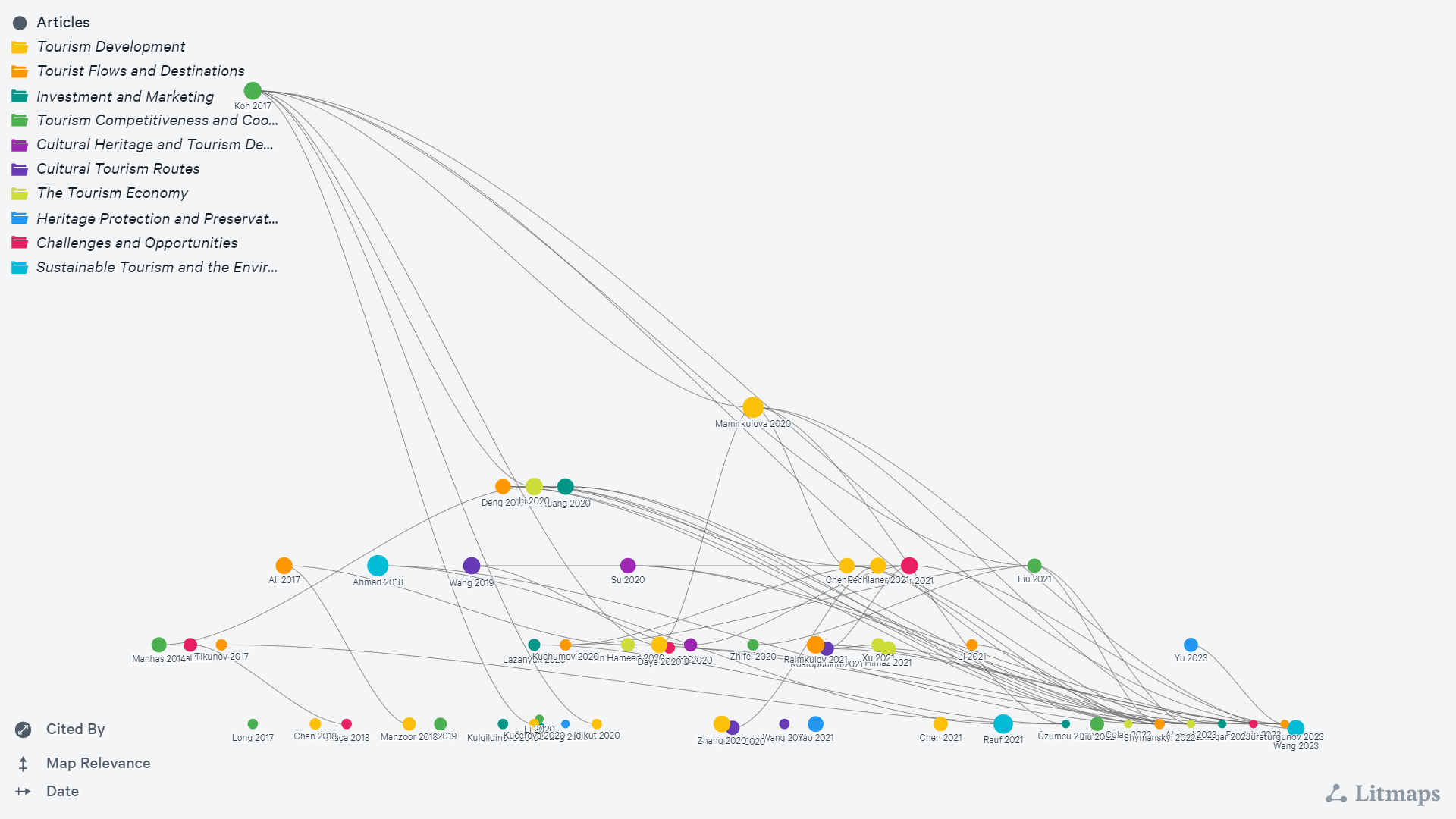
**

1. **Article positions, cited by (map relevance & date)**

**S4 Fig. Bibliometric map created by Litmaps software: each node (dot) represents a different academic article, and the lines between them indicate citations.** Source: edited by the authors
