# Supplementary material for: The Belt and Road Initiative’s impact on tourism and heritage along the Silk Roads: A systematic literature review and future research agenda
Source: PLoS One. 2024 Jul 18;19(7):e0306298. doi: 10.1371/journal.pone.0306298 (PMC11257252; doi:10.1371/journal.pone.0306298)
Supplement: S5 Fig — Source: edited by the authors. (DOCX) [file pone.0306298.s005.docx]

1. **Classification of the 56 selected studies by publisher**
2. **Classification of the 56 selected studies by journal**
3. **Classification of the 56 selected studies by date**
4. **Classification of the 56 selected studies by author**
5. **Classification of the 56 selected studies by geography**
6. **Classification of the 56 selected studies by research area**

**S5 Fig. Classification of 56 studies by publisher, journal, date, author, geography and research area.** Source: edited by the authors

These charts in (S5 Fig) provide detailed classifications of the 56 selected studies across various categories:

1. **Publishers:** The top publishers were MDPI with 11 papers (20%) and Taylor & Francis (Routledge) with 12 papers (22%).
2. **Journals:** The most common journals were *Sustainability* from MDPI with 10 papers (18%), *Service Industries Journal* from Taylor & Francis with 3 papers (6%), and *PLOS ONE* from Public Library of Science with 3 papers (6%).
3. **Date:** 42 out of 56 papers (75%) were published between 2020-2023, indicating a recent surge in research output.
4. **Authors:** 24 papers (43%) had Chinese first authors, while 32 papers (57%) had first authors of other nationalities.
5. **Geography:** 16 papers (29%) focused on China or China + other countries, while 40 papers (71%) covered global contexts or specific regions outside China.
6. **Research Areas:** 41 papers (73%) were on tourism topics, and 15 papers (27%) were on heritage topics.

The charts also provide a comprehensive view of the trends and focus areas in the selected studies, emphasizing the recent surge in publications and the research's diverse geographical and thematic scope.
