# Supplementary material for: The Belt and Road Initiative’s impact on tourism and heritage along the Silk Roads: A systematic literature review and future research agenda
Source: PLoS One. 2024 Jul 18;19(7):e0306298. doi: 10.1371/journal.pone.0306298 (PMC11257252; doi:10.1371/journal.pone.0306298)
Supplement: S1 Table — (DOCX) [file pone.0306298.s006.docx]

| **Section/topic** | **#** | **Checklist item** | **Reported on page #** |
| --- | --- | --- | --- |
| **TITLE** | | |  |
| Title | 1 | The report is identified as a systematic review. | 1 |
| **ABSTRACT** | | |  |
| Structured summary | 2 | The abstract outlines the scope, objectives, search strategy, study eligibility criteria, analysis methods, results, limitations, and conclusions of the systematic review. | 2 |
| **INTRODUCTION** | | |  |
| Rationale | 3 | The introduction discusses the background and significance of the China's Belt and Road Initiative (BRI) and Silk Roads, emphasizing the need to review emergent effects on tourism and heritage. | 3 and 4 |
| Objectives | 4 | The objectives are to evaluate the BRI's impact on tourism and heritage patterns along the Silk Roads, identify research gaps, and formulate a future research agenda. | 3 and 4 |
| **METHODS** | | |  |
| Protocol and registration | 5 | The review follows the PRISMA guidelines and has yet to be registered. | 5 and **S1** Table |
| Eligibility criteria | 6 | The review includes English language studies from 2013-2023 on the BRI's tourism and heritage impacts along the Silk Roads. | 7 |
| Information sources | 7 | The authors searched Scopus, Web of Science, and Google Scholar databases. | 7, **S2** Table and **S3** Table |
| Search | 8 | Search terms related to the BRI, Silk Roads, tourism, and heritage were used for the period from 2013 to 2023. | 7 |
| Study selection | 9 | The authors screened 1,007 items, excluded duplicates and any irrelevant studies, and included 56 items. | 7 and Fig.1 |
| Data collection process | 10 | A detailed review and analysis of selected articles was conducted through a structured methodology, including screening titles, abstracts, and content to identify themes. | 7 |
| Data items | 11 | The review focused on topics such as tourism development, cultural heritage preservation, destinations and infrastructure. | 7, 11, Table 2, **S4** Fig, Table 4 and **S5** Fig |
| Risk of bias in individual studies | 12 | The quality of the included studies was assessed using the Mixed Methods Appraisal Tool (MMAT) 2018 version, considering methodological transparency, theoretical consistency, and researcher positionality. | 7, 8, 11 and Table 1 |
| Summary measures | 13 | A qualitative thematic analysis was utilized. | 7 and 8 |
| Synthesis of results | 14 | Evidence was synthesized inductively into 2 key domains and 10 associated sub-themes. | 11 and Table 2 |

| **Section/topic** | **#** | **Checklist item** | **Reported on page #** |
| --- | --- | --- | --- |
| Risk of bias across studies | 15 | The authors assessed heterogeneity, disciplinary biases, generalizability, and sample size limitations across studies. | 7, 8, 11 and Table 1 |
| Additional analyses | 16 | Sensitivity analyses were conducted based on study quality. | 7, 8, 11, 19, 20, 24, 25, Table 1, **S2** Table and **S3** Table |
| **RESULTS** | | |  |
| Study selection | 17 | The review started with 1,007 initial records and ended with 56 studies ultimately included. | 11, Fig.1 and Table 2 |
| Study characteristics | 18 | An overview of author demographics, publication details, geographic distribution, etc., is provided. | 20, 24, Table 4 and **S5** Fig |
| Risk of bias within studies | 19 | The MMAT assessment revealed varying levels of methodological quality among the included studies. | 7, 8, 11, 19, 20, 24, 25 and Table 1 |
| Results of individual studies | 20 | Thematic findings across tourism and heritage impacts are presented, structured by 2 domains and 10 sub-domains. | 11 and Table 2 |
| Synthesis of results | 21 | The review summarizes the findings from the included studies' synthesis and identifies gaps and thematic concentrations in the current literature. | 14-18, 20, 24, 25 and Table 3 |
| Risk of bias across studies | 22 | The authors discussed multiplicities, disciplinary skews, and generalizability constraints across studies. | 7, 8, 11, 19, 20, 24, 25 and Table 1 |
| Additional analysis | 23 | A bibliometric analysis using Litmaps and a SWOT analysis detailing strengths, weaknesses, opportunities, and threats were conducted. | 18, **S4** Fig and Table 3 |
| **DISCUSSION** | | |  |
| Summary of evidence | 24 | The discussion highlighted disciplinary, geographic, and linguistic gaps needing redress. | 20, 24, 25, Table 4 and **S5** Fig |
| Limitations | 25 | The authors acknowledged linguistic selectivity, imbalance, data constraints, and political fluctuations as limitations. | 25 |
| Conclusions | 26 | The review emphasizes the BRI's potential impact on tourism and heritage, advocating for inclusion, cooperation, sustainability, and further research. | 25 and 26 |
| **FUNDING** | | |  |
| Funding | 27 | The research was supported by Chinese national and provincial grants. | 27 |

*From:*  Moher D, Liberati A, Tetzlaff J, Altman DG, The PRISMA Group (2009). Preferred Reporting Items for Systematic Reviews and Meta-Analyses: The PRISMA Statement. PLoS Med 6(7): e1000097. doi:10.1371/journal.pmed1000097

For more information, visit: **www.prisma-statement.org**.
