# Supplementary material for: The Belt and Road Initiative’s impact on tourism and heritage along the Silk Roads: A systematic literature review and future research agenda
Source: PLoS One. 2024 Jul 18;19(7):e0306298. doi: 10.1371/journal.pone.0306298 (PMC11257252; doi:10.1371/journal.pone.0306298)
Supplement: S2 Table — Source: edited by the authors. (DOCX) [file pone.0306298.s007.docx]

**S2 Table. The journals in which the 56 selected studies were published were identified based on their standing in SCImago Journal Rank (SJR) and inclusion in the Scopus database (Elsevier) (38 articles, 68%).** Source: edited by the authors

| **Publisher** | **Journals** | **Journal rank, subject area and category** | **H-Index** | **Number of Studies** | **Web of Science (WoS) included** |
| --- | --- | --- | --- | --- | --- |
| 1. Multidisciplinary Digital Publishing Institute (MDPI)   (11) Studies  (20%) | 1. Sustainability   ISSN: 2071-1050  <https://www.mdpi.com/journal/sustainability> | 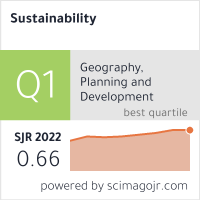 | 136 | 10  ~ (18%) | Yes  SCI/SSCI |
|  | 1. Land   ISSN: 2073-445X  <https://www.mdpi.com/journal/land> | 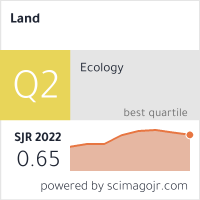 | 42 | 1  ~ (2%) | Yes  SSCI |
| 1. Taylor & Francis (Routledge)   (12) Studies  ~ (22%) | 1. Current Issues in Tourism   ISSN: 1368-3500  <https://www.tandfonline.com/journals/rcit20> | 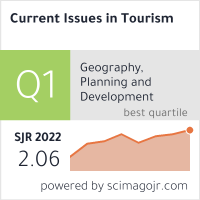 | 94 | 1  ~ (2%) | Yes  SSCI |
|  | 1. Service Industries Journal   ISSN: 0264-2069  <https://www.tandfonline.com/journals/fsij20> | 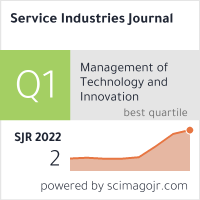 | 76 | 3  ~ (6%) | Yes  SSCI |
|  | 1. Asia Pacific Journal of Tourism Research   ISSN: 1094-1665  <https://www.tandfonline.com/journals/rapt20> | 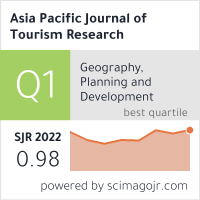 | 53 | 1  ~ (2%) | Yes  SSCI |
|  | 1. Territory, Politics, Governance   ISSN: 2162-2671  <https://www.tandfonline.com/journals/rtep20> | 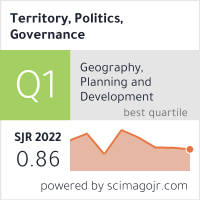 | 31 | 1  ~ (2%) | Yes  SSCI |
|  | 1. International Journal of Cultural Policy   ISSN: 1028-6632  <https://www.tandfonline.com/toc/gcul20/current> | 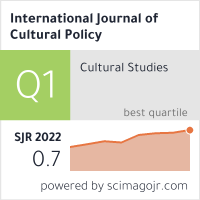 | 52 | 1  ~ (2%) | Yes  SSCI/ AHCI |
|  | 1. International Journal of Heritage Studies   ISSN: 1352-7258  <https://www.tandfonline.com/journals/rjhs20> | 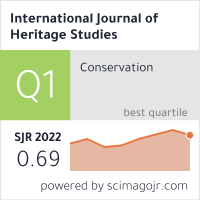 | 56 | 1  ~ (2%) | Yes  SSCI/ AHCI |
|  | 1. World Archaeology   ISSN: 0043-8243  <https://www.tandfonline.com/journals/rwar20> | 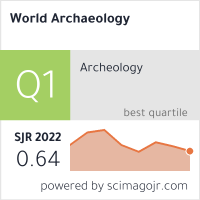 | 76 | 1  ~ (2%) | Yes  AHCI |
|  | 1. Journal of China Tourism Research   ISSN: 1938-8160  <https://www.tandfonline.com/journals/rwar20> | 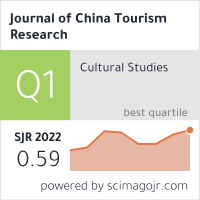 | 26 | 2  ~ (4%) | Yes  ESCI |
|  | 1. Journal of Quality Assurance in Hospitality and Tourism   ISSN: 1528-008X  <https://www.tandfonline.com/journals/wqah20> | 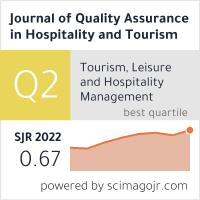 | 36 | 1  ~ (2%) | Yes  ESCI |
| 1. Elsevier   (4) Studies  ~ (7%) | 1. Journal of Destination Marketing and Management   ISSN: 2212-571X  <https://www.sciencedirect.com/journal/journal-of-destination-marketing-and-management> | 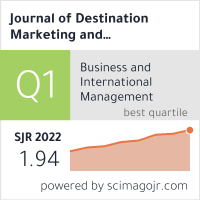 | 63 | 1  ~ (2%) | Yes  SSCI |
|  | 1. Tourism Management Perspectives   ISSN: 2211-9744  <https://www.sciencedirect.com/journal/tourism-management-perspectives> | 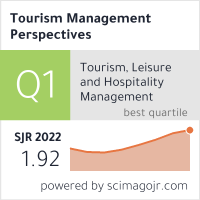 | 69 | 1  ~ (2%) | Yes  SSCI |
|  | 1. Global Ecology and Conservation   ISSN: 2351-9894  <https://www.sciencedirect.com/journal/global-ecology-and-conservation> | 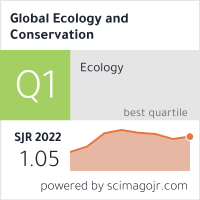 | 57 | 1  ~ (2%) | Yes  SCI |
|  | 1. Procedia - Social and Behavioral Sciences   ISSN: 18770428  <https://www.sciencedirect.com/journal/procedia-social-and-behavioral-sciences> | 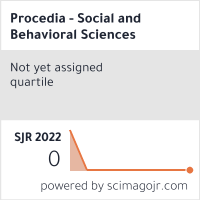 | 67 | 1  ~ (2%) | No |
| 1. SAGE Publications   (3) Studies  ~ (6%) | 1. Tourism Economics   ISSN: 1354-8166  <https://journals.sagepub.com/home/teu> | 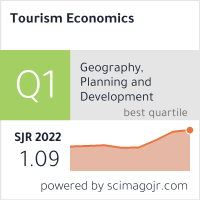 | 69 | 2  ~ (4%) | Yes  SSCI |
|  | 1. Evaluation Review   ISSN: 0193-841X  <https://journals.sagepub.com/home/erx> | 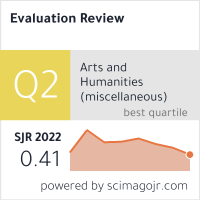 | 59 | 1  ~ (2%) | Yes  SSCI |
| 1. Public Library of Science   (3) Studies  ~ (6%) | 1. PLOS ONE   ISSN: 1932-6203  <https://journals.plos.org/plosone/> | 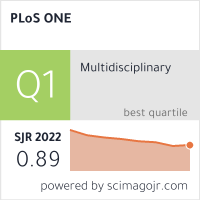 | 404 | 3  ~ (6%) | Yes  SCI |
| 1. Springer   (1) Study  ~ (2%) | 1. Journal of Archaeological Research   ISSN: 1059-0161  <https://link.springer.com/journal/10814> | 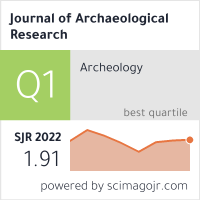 | 60 | 1  ~ (2%) | Yes  SSCI/ AHCI |
| 1. Walter de Gruyter GmbH   (1) Study  ~ (2%) | 1. Zeitschrift fur Wirtschaftsgeographie   ISSN: 22748-1956  <https://www.degruyter.com/journal/key/zfw/html> | 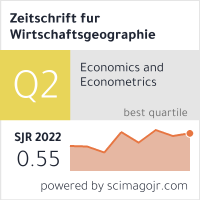 | 19 | 1  ~ (2%) | Yes  SSCI |
| 1. Lomonosov Moscow State University, Faculty of Geography   (1) Study  ~ (2%) | 1. Geography, Environment, Sustainability   ISSN: 2071-9388  <https://ges.rgo.ru/jour> | 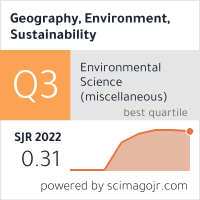 | 13 | 1  ~ (2%) | No |
| 1. Hindawi Publishing Corporation   (1) Study  ~ (2%) | 1. Discrete Dynamics in Nature and Society   ISSN: 1026-0226  <https://www.hindawi.com/journals/ddns/> | 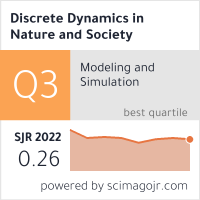 | 46 | 1  ~ (2%) | Yes  SCI |
| 1. Oxford University Press   (1) Study  ~ (2%) | 1. Chinese Journal of Comparative Law   ISSN: 2050-4802  <https://academic.oup.com/cjcl?login=false> | 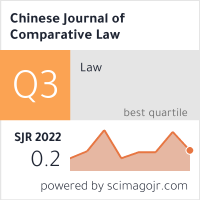 | 9 | 1  ~ (2%) | Yes  ESCI |

These tables provide a detailed breakdown of the journals where the 56 selected studies were published, classified based on their standing in the SCImago Journal Rank (SJR) and inclusion in the Scopus database, as well as their Google Scholar standing. These tables include specific information such as the journal's rank, H-index, and the number of studies published in each journal. Additionally, the tables list the publishers of these journals and indicate whether they are included in the Web of Science (WOS). This detailed classification offers a comprehensive view of the academic impact and reach of the journals involved in these studies.

**S2 Table Highlights:**

- 38 out of 56 studies (68%) were published in journals indexed in Scimago Journal Rank (SJR) and Scopus.

- Top publishers were MDPI (20% of studies), Taylor & Francis (22%), Elsevier (7%), and Sage (6%).

- Leading journals included "Sustainability" (MDPI), "Service Industries Journal" (Taylor & Francis), and "Tourism Economics" (Sage).

- 36 of the journals were indexed in Web of Science (WOS).
