# Supplementary material for: The Belt and Road Initiative’s impact on tourism and heritage along the Silk Roads: A systematic literature review and future research agenda
Source: PLoS One. 2024 Jul 18;19(7):e0306298. doi: 10.1371/journal.pone.0306298 (PMC11257252; doi:10.1371/journal.pone.0306298)
Supplement: S3 Table — Source: edited by the authors. (DOCX) [file pone.0306298.s008.docx]

**S3 Table. The journals in which the 56 selected studies were published were identified based on their standing in Google Scholar (18 studies, 32%).** Source: edited by the authors

| **Publisher** | **Journals** | **Type** | **Number of Studies** | **Web of Science (WoS) included** |
| --- | --- | --- | --- | --- |
| 1. The Canadian Center of Science and Education (CCSE)   (2) Studies  ~ (4%) | 1. International Business Research   ISSN: 1913-9004  <https://ccsenet.org/journal/index.php/ibr/about> | Article | 1  ~ (2%) | No |
|  | 1. Journal of Management and Sustainability   ISSN: 1925-4725  <https://www.ccsenet.org/journal/index.php/jms/about> | Article | 1  ~ (2%) | No |
| 1. Sciendo fa parte della società De Gruyter   (1) Study  ~ (2%) | 1. Confrontation and Cooperation: 1000 Years of Polish-German-Russian Relations   eISSN: 2391-5536  <https://sciendo.com/it/journal/CONC> | Article | 1  ~ (2%) | No |
| 1. Scientific Research Publishing Inc   (1) Study  ~ (2%) | 1. Advances in Applied Sociology   ISSN: 2165-4328  <https://www.scirp.org/journal/journalarticles?journalid=1002> | Article | 1  ~ (2%) | No |
| 1. Atlantis Press   (7) Studies  ~ (13%) | 1. Advances in Economics, Business and Management Research   ISSN (Online): 2352-5428  <https://www.atlantis-press.com/proceedings/series/aebmr> | Conference  proceeding | 3  ~ (6%) | NO |
|  | 1. Advances in Social Science, Education and Humanities Research   ISSN (Online): 2352-5398  <https://www.atlantis-press.com/proceedings/series/assehr> | Conference  proceeding | 4  (7%) | NO |
| 1. World Center of Innovation Research and Publication   (1) Study  ~ (2%) | 1. New Trends and Issues Proceedings on Humanities and Social Sciences   ISSN: 2547-8818  <https://un-pub.eu/ojs/index.php/pntsbs> | Conference  proceeding | 1  ~ (2%) | No |
| 1. IOP Science   (1) Study  ~ (2%) | 1. IOP Conference Series: Earth and Environmental Science   ISSN: 1755-1315  <https://iopscience.iop.org/journal/1755-1315> | Conference  proceeding | 1  ~ (2%) | NO |
| 1. IGI Global   (1) Study  ~ (2%) | 1. Handbook of Research on Current Trends in Asian Economics, Business, and Administration   ISBN13: 9781799884866  <https://www.igi-global.com/book/handbook-research-current-trends-asian/270868> | Book chapter | 1  ~ (2%) | No |
| 1. Springer   (4) Study  ~ (7%) | 1. China and the New Silk Road   ISBN: 978-3-030-43398-7  <https://link.springer.com/book/10.1007/978-3-030-43399-4> | Book chapter | 2  ~ (4%) | No |
|  | 1. Normative Readings of the Belt and Road Initiative   ISBN: 978-3-319-78017-7  <https://link.springer.com/book/10.1007/978-3-319-78018-4> | Book chapter | 1  ~ (2%) | No |
|  | 1. Cities’ Vocabularies and the Sustainable Development of the Silkroads   ISBN: 978-3-031-31026-3  <https://link.springer.com/book/10.1007/978-3-031-31027-0> | Book chapter | 1  ~ (2%) | No |

These tables provide a detailed breakdown of the journals where the 56 selected studies were published, classified based on their standing in the SCImago Journal Rank (SJR) and inclusion in the Scopus database, as well as their Google Scholar standing. These tables include specific information such as the journal's rank, H-index, and the number of studies published in each journal. Additionally, the tables list the publishers of these journals and indicate whether they are included in the Web of Science (WOS). This detailed classification offers a comprehensive view of the academic impact and reach of the journals involved in these studies.

**S3 Table Highlights:**

- 18 out of 56 studies (32%) were published in journals found in Google Scholar.

- Publishers included CCSE (4% of studies), Sciendo (2%), and Atlantis Press (13%).

- Publication types included articles, conference proceedings, and book chapters.

- None of the journals were indexed in Web of Science (WOS).
